# Disease-induced changes in plant microbiome assembly and functional adaptation

**DOI:** 10.1186/s40168-021-01138-2

**Published:** 2021-09-15

**Authors:** Min Gao, Chao Xiong, Cheng Gao, Clement K. M. Tsui, Meng-Meng Wang, Xin Zhou, Ai-Min Zhang, Lei Cai

**Affiliations:** 1 State Key Laboratory of Mycology, Institute of Microbiology, Chinese Academy of Sciences, Beijing, 100101 China; 2 College of Life Sciences, University of Chinese Academy of Sciences, Beijing, 100049 China; 3 State Key Laboratory of Urban and Regional Ecology, Research Center for Eco-Environmental Sciences, Chinese Academy of Sciences, 100085 Beijing, China; 4 Department of Pathology, Sidra Medicine, Doha, Qatar; 5 Department of Pathology and Laboratory Medicine, Weill Cornell Medicine-Qatar, Doha, Qatar; 6 Division of Infectious Diseases, Faculty of Medicine, University of British Columbia, Vancouver, BC Canada; 7 Pepper Research Institute, Guizhou Provincial Academy of Agricultural Sciences, 550009 Guiyang, China

**Keywords:** *Fusarium* wilt disease, Compartment, Microbiome assembly, Microbial network, Beneficial microbe, Metagenomics, Chili pepper

## Abstract

**Background:**

The plant microbiome is an integral part of the host and increasingly recognized as playing fundamental roles in plant growth and health. Increasing evidence indicates that plant rhizosphere recruits beneficial microbes to the plant to suppress soil-borne pathogens. However, the ecological processes that govern plant microbiome assembly and functions in the below- and aboveground compartments under pathogen invasion are not fully understood. Here, we studied the bacterial and fungal communities associated with 12 compartments (e.g., soils, roots, stems, and fruits) of chili pepper (*Capsicum annuum* L.) using amplicons (16S and ITS) and metagenomics approaches at the main pepper production sites in China and investigated how *Fusarium* wilt disease (FWD) affects the assembly, co-occurrence patterns, and ecological functions of plant-associated microbiomes.

**Results:**

The amplicon data analyses revealed that FWD affected less on the microbiome of pepper reproductive organs (fruit) than vegetative organs (root and stem), with the strongest impact on the upper stem epidermis. Fungal intra-kingdom networks were less stable and their communities were more sensitive to FWD than the bacterial communities. The analysis of microbial interkingdom network further indicated that FWD destabilized the network and induced the ecological importance of fungal taxa. Although the diseased plants were more susceptible to colonization by other pathogenic fungi, their below- and aboveground compartments can also recruit potential beneficial bacteria. Some of the beneficial bacterial taxa enriched in the diseased plants were also identified as core taxa for plant microbiomes and hub taxa in networks. On the other hand, metagenomic analysis revealed significant enrichment of several functional genes involved in detoxification, biofilm formation, and plant-microbiome signaling pathways (i.e., chemotaxis) in the diseased plants.

**Conclusions:**

Together, we demonstrate that a diseased plant could recruit beneficial bacteria and mitigate the changes in reproductive organ microbiome to facilitate host or its offspring survival. The host plants may attract the beneficial microbes through the modulation of plant-microbiome signaling pathways. These findings significantly advance our understanding on plant-microbiome interactions and could provide fundamental and important data for harnessing the plant microbiome in sustainable agriculture.

**Video abstract**

**Supplementary Information:**

The online version contains supplementary material available at 10.1186/s40168-021-01138-2.

## Background

Plants and the associated microbiomes have co-evolved for more than 400 millions of years and form a “holobiont” within which plant-microbiome interactions play essential roles in many aspects of host functionality and fitness [1–5], including nutrient acquisition [6–8], abiotic stress tolerance [9], and disease suppression [10, 11]. Consequently, manipulation of the plant microbiome is increasingly considered as an environmentally sustainable approach to protect the plant from disease and to promote agricultural production [4, 12]. Uncovering the fundamental ecological patterns that govern the assembly, co-occurrence patterns, and functions of plant-associated microbiomes and how do plant hosts modulate their microbiomes under external stress are prerequisite for harnessing plant microbiomesmicrobiome to enhance plant health and to maximize crop production.

Plant microbiome assembly is shaped by multiple biotic and abiotic factors, such as host selection (e.g., plant compartment and host genetics) [13–15], climate, and soil type [16, 17]. Apart from the host selection and herbivorous insects [18], pathogen invasion [19, 20] is one of the most influential biotic stress affecting plant microbiome assembly. Accumulating studies on wheat [21, 22], sugar beet [11], and *Arabidopsis thaliana* [23] have shown that the roots of pathogen-infected plants can attract beneficial microbes for rescue or protect future generations (i.e., “cry for help” strategy). The host plants can attract beneficial microbes by emitting volatile organic compounds (VOCs) or modifying synthesis and secretion of particular root exudates [18, 23–26]. The beneficial microbes acting as keystone taxa of the plant microbiome could contribute to plant disease suppression by priming the plant immune system, excreting antibiotic compounds, and competing resources with pathogen [27]. In addition to roots, our understanding of whether other plant organs (e.g., stems and fruits) use the similar strategy to seek microbial benefits under pathogen infection is still largely unknown.

A growing body of experimental and observational literature has provided evidence that the rhizosphere is a critical zone of the plant and its microbiomes are closely related to plant performance [9, 13, 25, 28, 29]. On the other hand, the phyllosphere microbiome, i.e., microbes inhabiting the aerial parts of the plant, may play essential but often overlooked roles in plant health, productivity, and ecosystem function [30–33]. Several recent studies indicated that infections with aboveground pathogens alter the plant’s rhizosphere microbial community [23, 26]. Further, the rhizosphere microbiome acting as the seed banks of the phyllosphere microbiome plays a key role in determining the aboveground productivity and health [34, 35]. Bai et al. [36] established leaf- and root-derived microbiota cultures in *Arabidopsis thaliana* and found an extensive taxonomic overlap between them. Collectively, above studies indicate that the below- and aboveground microbiomes of plants are systematically linked. To date, however, most related studies often focused on the rhizosphere or phyllosphere microbiomes, and a systematic understanding of microbiome structure and functions across the rhizosphere, phyllosphere, and endosphere under pathogen invasion remains unclear. Also, microbial community assembly is largely influenced by the cooperative and competitive interactions among the myriad microbial members that perform functions for plant health as a whole [37, 38]. Co-occurrence network analysis has been increasingly used to infer the potential microbial interconnections and interrogate the community stability based on topological properties [17, 39]. Based on theoretical modeling and simulation data, microbial networks having the properties of greater modularity, lower positive correlations among members, and higher negative correlations among members are more stable [39–41]. Nevertheless, our understanding on the potential interactions within complex plant-associated microbiomes, and how they respond to pathogen invasion, remains scant.

*Fusarium* wilt disease (FWD) is often caused by the *Fusarium oxysporum* species complex [42], a classical soil-borne disease that attacks a wide variety of economically important crops [43–45], including banana [46, 47], watermelon, and *Solanaceae* plants (e.g., tomato, eggplant, and chili pepper). The pathogen enters through the root and interferes with the plant’s water-conducting vessels, leading to brown vascular bundle formation and wilt symptoms. Chili pepper (*Capsicum annuum* L.) is one of the major agricultural crops worldwide, and China accounts for over 50% of the world’s chili pepper production according to the Food and Agriculture Organization. FWD in pepper is caused by *F. oxysporum* f. sp. *capsici* [42, 48] and leads to severe production losses annually.

Plants consist of different organs, which are classified as either vegetative (the root, stem, and leaf) or reproductive (the fruit, flower, and seed), each of which has specific functions. Since a plant may enhance offspring fitness [22, 26], we hypothesized that a disease would more severely affect vegetative organs than reproductive organs, and that the infected plant recruits protective microbes to suppress the growth of pathogen. Further, considering that fungal communities are more responsive to vegetation change than bacterial communities [49], and that fungi are the first consumers of the belowground plant-derived carbon [50–52], we also expected that the fungal communities of chili pepper are more sensitive to FWD than bacterial communities. Finally, considering much evidences linking the taxonomic composition and ecological function [30, 53–55], we hypothesized that the disease-induced changes in taxonomic composition influence the functional adaptation of the microbiome. To test these hypotheses, we here investigated the effect of FWD on chili pepper microbiomes in Guizhou, China, where FWD incidence is high and chili pepper is an important crop. Using a pepper–FWD system, we aimed to explore the taxonomic and functional differences between the microbiomes of healthy and diseased plants using amplicon (both bacterial and fungal) and metagenomic sequencing. We also compared the networks of healthy and diseased plant microbiomes to offer insights into the stability of communities as well as microbes that tend to co-occur with one another.

## Materials and methods

### Sampling

All samples were collected in the main chili pepper production fields in Huishui (25° 48′ 41″ N, 106° 31′ 24″ E) and Guiyang (26° 29′ 31″ N, 106° 39′ 16″ E; 92.1 km apart), in Guizhou province, southwest China. The two sites are located in a subtropical monsoon climate zone, with the same annual mean temperature of 15.8 °C, and annual mean precipitation of 1213.4 mm and 1259.8 mm, respectively. The pepper cultivars used were the same as those planted by local farmers (i.e., line pepper), with Changla No. 8 at Huishui and N1713 at Guiyang, respectively. Mature peppers were sampled in August 2018 at both sites. At each site, pepper plants that displayed no wilt symptoms and tested pathogen-negative were classified as healthy; plants that showed wilt, brown vascular bundle symptoms and tested pathogen-positive (confirmed by morphological and molecular data; the primers are listed in Table S1) were classified as diseased (Fig. S1). Three replicates of healthy and diseased plants were collected from three adjacent plots at each site. Each replicate consisted of a composite sample obtained by mixing three individual samples. While collecting a plant sample, a bulk soil sample was collected 20 cm away from the root, at a depth of 0–15 cm. Rhizosphere soil (defined as the soil that adheres to the root) was collected from the root by manually shaking. The plant samples, and the corresponding rhizosphere and bulk soil of each plant, were transported to the laboratory on dry ice and stored at −80 °C until further experiment.

### DNA extraction and amplicon sequencing

Root and fruit samples were fractionated into the episphere and endosphere compartments, representing microbes residing on the root and fruit surface or inside the organ, respectively. For microbial DNA extraction from the episphere, 10–20 g fruits or 3–5 g roots (obtained after carefully removing large chunks of soil from the roots using sterile cotton swabs) were placed in sterile bottles or polystyrene tubes containing release buffer (0.1 M potassium phosphate, 0.1% glycerol, and 0.15% Tween 80, pH 7.0; 150 ml for fruit analysis and 35 ml for root analysis) and sonicated at 40 kHz for 1 min. The samples were then shaken for 4 min at 200 rpm on a shaker [14]. This procedure was repeated twice. The wash liquid was then filtered through a 0.22-μm nitrocellulose membrane filter (BOJIN, Germany). The filters, containing episphere microorganisms, were stored at –80 °C before DNA extraction.

For microbial DNA extraction from the endosphere, approximately 5 g fruits or roots were treated as above to dislodge the epiphytes. Then, the plant material was rinsed with 70% ethanol for 5 min, 5.25% sodium hypochlorite solution for 5 min, and 70% ethanol for 30 s and finally washed with sterile H_2_O, five times, for surface sterilization. The treated fruit and root samples were ground using sterile mortar and pestle and frozen at –80 °C. The pepper stem samples were divided into the upper stem section, middle stem section, and bottom stem section, with each section additionally divided into the epidermis and xylem, accordingly (Fig. S2). The epidermis and xylem fractions were ground using sterile mortar and pestle with liquid nitrogen. Total DNA was extracted from the samples using FastDNA SPIN Kit for Soil (MP Biomedicals, Solon, USA) following the manufacturer’s instructions.

**Fig. 1:**
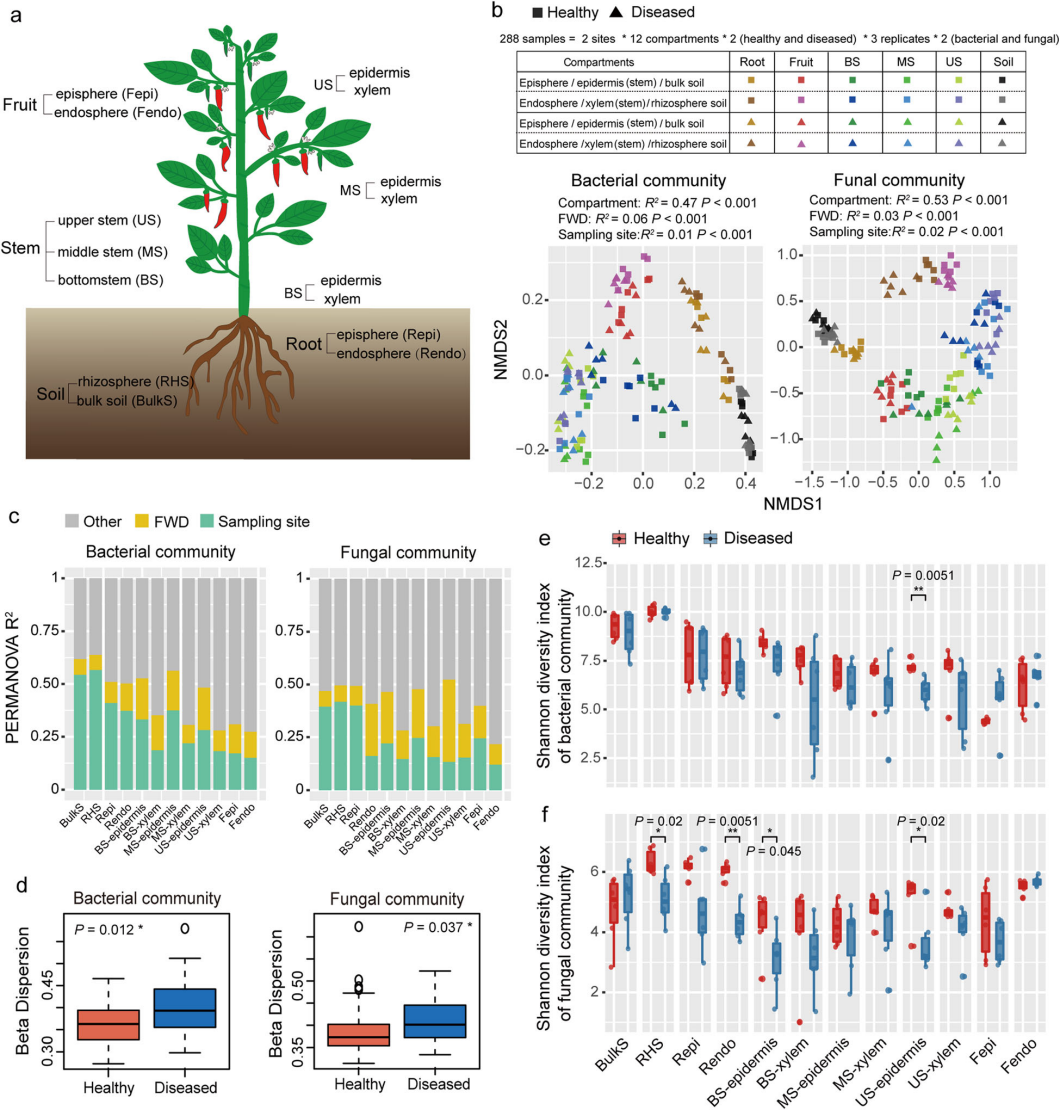
Assembly of pepper bacterial and fungal communities. **a** Diagram of a pepper plant, and the below- and aboveground compartments, including the soil, root, stem, and fruit. **b** Non-metric multi-dimensional scaling (NMDS) ordinations of Bray–Cutis dissimilarity matrices with permutational analysis of variance (PERMANOVA), showing significant association of the bacterial (left) and fungal (right) community composition with, in the order of importance, the compartment (*R*^2^ = 0.47 for bacteria and *R*^2^ = 0.53 for fungi), *Fusarium* wilt disease (FWD, *R*^2^ = 0.06 and 0.03, respectively), and sampling site (*R*^2^ = 0.01 and 0.02, respectively). **c** Contribution of FWD and sampling site to the variation of bacterial (left) and fungal (right) communities in a single compartment, based on PERMANOVA. FWD explains the higher variation of fungal community than that of the bacterial community in most compartments. **d** Beta-dispersion analysis (based on Bray–Cutis dissimilarity) indicating higher dissimilarity of the bacterial (left) and fungal (right) communities in diseased plants than in healthy plants .**e–f** Shannon diversity indices of bacterial and fungal communities in the 12 compartments of healthy (red) and diseased (blue) plants

Overall, each plant sample was divided into 12 compartments: the bulk soil (BulkS), rhizosphere soil (RHS), root episphere (Repi) and endosphere (Rendo), bottom stem epidermis (BS-epidermis) and xylem (BS-xylem), middle stem epidermis (MS-epidermis) and xylem (MS-xylem), upper stem epidermis (US-epidermis) and xylem (US-xylem), and fruit episphere (Fepi) and endophere (Fendo) (Fig. 1a and Fig. S2).

The V5–V6 region of bacterial 16S rRNA gene (799F/1115R) [14, 56] and the fungal ITS2 region (fITS7/ITS4) [17, 57, 58] were amplified (see Additional file 1 for details; primer sequences and PCR amplification conditions are shown in Table S1). Amplicon libraries were sequenced on the Illumina HiSeq2500 platform (MEGIGENE Biological Company, Guangdong, China).

### Analysis of amplicon sequencing data

The bacterial 16S rRNA gene and fungal ITS sequences were processed using USEARCH v10.0 [59] and QIIME v1.9.1 [60]. Briefly, the primer sequences and low-quality read with scores below Q30 were trimmed. Paired 16S and ITS reads were merged into a single sequence. ITS reads were trimmed to 200 bp and quality-filtered (maximum expected error 0.5). Biological reads were identified at 100% sequence similarity using unoise3 [61] with default parameters. Taxonomic assignment was performed using SILVA reference database (v12_8) [62] and UNITE database (v7.0) [63] for bacteria and fungi, respectively. Bacterial zero-radius operational taxonomic units (ZOTUs) assigned to the chloroplast, mitochondrion, or viridiplantae, as well as fungal ZOTUs, assigned to plant or protist were removed. ZOTUs represented by less than two sequences were also removed to avoid possible bias.

Cumulative sum scaling (CSS) was used as a normalization method for bacterial and fungal beta-diversity analyses [64]. Alpha diversity and beta-diversity indices of bacterial and fungal communities were calculated in QIIME v1.91 (using single_rarefaction.py, alpha_diversity.py, and beta_diversity.py scripts); the bacterial and fungal ZOTU tables were rarefied to 10,250 and 5005 reads for alpha diversity index estimates, respectively. As in previous studies [46, 65], the core taxa of healthy and diseased plant microbiomes were defined as ZOTUs present in 100% samples of healthy and diseased plants, respectively. Fungal ZOTUs were assigned into functional guilds using the online application FUNGuild (http://www.stbates.org/guilds/app.php) [66]. Confidence ranking of “Highly probable” and “Probable” was retained for high accuracy.

### Metagenomic sequencing workflow and data analysis

Based on the amplicon sequencing data, pepper samples of the upper stem epidermis and root endosphere collected at Huishui site were selected for metagenomic sequencing and characterization. Twelve DNA samples were sequenced as 150-bp paired-end reads using an Illumina NovaSeq 6000 instrument (Majorbio Bio-pharm Technology, Shanghai, China). Approximately 20 GB clean data were obtained for each DNA sample. To remove host-derived sequences, Bowtie2 v2.4.1 [67] was used to build a host genome database (*C. annuum* cultivar Zunla-1, NCBI reference sequence ASJU00000000.1) and the metagenomic data were then mapped against the host genome database. The remaining reads were assembled by using MEGAHIT v1.2.9 [68], predicted based on contigs by using Prokka v1.14.5 [69], and clustered at 0.95 similarity threshold by using CD-HIT v4.8.1 to generate a non-redundant gene catalog. Functional annotation was performed by eggnog-mapper v1.0.3 [70] using DIAMOND comparison [71] and eggNOG databases (v5.0) [72]. The annotation results were reorganized into Kyoto Encyclopedia of Genes and Genomes (KEGG) Orthology (KO) profiles [73], Clusters of Orthologous Group of proteins (COG) functional categories [74], and CAZymes (CAZ) [75]. The antibiotic resistance genes were detected and reorganized using ResFams [76]. Functional diversity was calculated using QIIME v1.91 (using single_rarefaction.py, alpha_diversity.py, and beta_diversity.py script), and the effect of FWD on functional dissimilarity was tested using betadisper function in vegan package in R [77]. Differential abundance of the functional genes between healthy and diseased plant microbiomes was explored via LDA effect size (LEfSe) analysis (Galaxy web application, http://huttenhower.sph.harvard.edu/galaxy/) [78]. Taxonomic classification of metagenomic sequence data was inferred using Kraken 2 [79], which generates *k*-mer matches to achieve high accuracy with fast classification speed. Species abundance was calculated using Bracken [80], a companion program of Kraken 2.

### Statistical analysis

Alpha diversity indices (e.g., Shannon index and Chao1 index) were calculated using QIIME v1.91 (alpha_diversity.py). The differences among samples of each compartment from healthy and diseased plants were tested using Wilcoxon rank-sum tests. Linear-mixed models (LMMs) were employed to identify the major drivers of alpha diversity index and community composition (phylum and class levels). The variable strength was compared using type II analysis of variance (ANOVA) and *R*^*2*^ was calculated for the model [81]. Bray–Curtis distance matrices were calculated and visualized using non-metric multi-dimensional scaling (NMDS) ordinations to assess the bacterial and fungal community beta-diversity. Permutational multivariate analysis of variance (PERMANOVA) statistical tests were performed to determine the effects of different factors on the community dissimilarity using “*adonis*” in vegan R package [82], with 1999 permutations and using Bray–Curtis distance matrix as an input. PERMANOVA was also implemented to test the impacts of FWD and sampling site in single compartments. To calculate beta-dispersion, betadisper function in vegan R package, which is a multivariate analog of Levene’s test for homogeneity of variances, was performed. Differential abundance analysis between healthy and diseased plant microbiomes was calculated using EdgeR’s generalized linear model (GLM) approach in “edgeR” R package [83], using a trimmed mean of M-values (TMM) normalization method and a threshold of significance at *P* < 0.001.

### Co-occurrence network analysis

Co-occurrence patterns were reconstructed by calculating multiple abundance correlations based on genus-level matrices using co-occurrence network (CoNet) app in Cytoscape [84]. A co-occurrence was considered to be robust if the Spearman’s correlation coefficient (ρ) was > 0.70 and *P* < 0.05. The *P* values were adjusted using Benjamini–Hochberg procedure to minimize false-positive signals [85]. The networks were visualized using the interactive platform Gephi [86]. Nodes represent the individual microbial genera, and edges represent the pairwise correlations between the nodes in the microbiome network, indicating biologically or biochemically meaningful interactions.

The calculated topological characteristics of bacterial and fungal networks included the numbers of co-occurrence (positive) and mutual exclusion (negative) correlations, average path length, network diameter, average clustering coefficient, average connectivity, and modularity. The roles of individual nodes were determined based on topologicalfeatures of degree and closeness centrality [15]. The hub taxa in each network were identified as the top 10 nodes with the highest degree and closeness centrality. Network stability was measured by the proportion of negative or positive correlations and the modularity [17, 39, 40].

## Results

### FWD affects pepper microbiome assembly

In total, 8,672,206 bacterial 16S rRNA and 7,677,988 fungal ITS high-quality reads were obtained from 144 samples. These reads were sorted into 14,976 bacterial ZOTUs and 4277 fungal ZOTUs. To examine the dimensions in which the factors that shape the pepper microbiome, we assessed the relative contribution of multiple factors in terms of plant compartment, FWD, and sampling site in shaping the microbial communities. NMDS ordinations and PERMANOVA analysis revealed the greatest effect on the total microbiome exerted by the compartment (*R*^*2*^ = 0.47 for bacteria and *R*^*2*^ = 0.53 for fungi, *P* < 0.001 for both), followed by FWD (*R*^*2*^ = 0.06 for bacteria and *R*^*2*^= 0.03 for fungi, *P* =< 0.001 for both), and the sampling site (*R*^*2*^= 0.01 for bacteria and *R*^*2*^= 0.02 for fungi, *P* < 0.001 for both) (Fig. 1b and Table S2). FWD explained a higher proportion of variation of the fungal community than that of the bacterial community in the root endosphere, bottom stem epidermis, middle stem epidermis and xylem, upper stem epidermis and xylem, and fruit episphere (Fig. 1c, Table S3, and Table S4). Notably, FWD affected the fungal community in the pepper fruit to a lesser extent than it affected the community in the stem and root (root/stem/fruit *R*^*2*^: 0.17/0.22/0.13, on average, respectively; Fig. 1c and Table S4). Similarly, the impact of FWD on the bacterial community was stronger in the stem than in the fruit (stem/fruit *R*^*2*^: 0.16/0.13, on average, respectively; Fig. 1c and Table S3). For the stem, the effect of FWD on both bacterial and fungal communities was more pronounced in the epidermis (bacteria/fungi, *R*^*2*^: 0.19/0.29, on average) than in the xylem (bacteria/fungi, *R*^*2*^: 0.12/0.145, on average) (Fig. 1c, Table S3 and Table S4). In all compartments, FWD most affected the fungal community in the upper stem epidermis and root endosphere (*R*^*2*^ = 0.39, *P* =< 0.001 in the upper stem epidermis; and *R*^*2*^ = 0.25, *P* =< 0.001 in the root endosphere) (Fig. 1c and Table S4). By contrast, sampling site explained higher proportion of variation of the bacterial communities than FWD in most compartments (Fig. 1c and Table S3). In addition, both the bacterial (*P* = 0.012) and fungal communities (*P* = 0.037) in diseased plants were more variable than those in healthy plants, as determined based on beta-dispersion using Bray–Curtis dissimilarity (Fig. 1d). For each compartment, bacterial communities were more variable in the diseased plants than the healthy plants in the bulk soil, root endosphere, bottom stem epidermis and xylem, upper stem epidermis, and fruit episphere (Table S5). Fungal communities were more variable in the diseased plants than the healthy plants in the bulk soil, rhizosphere soil, middle stem xylem, upper stem epidermis, and fruit episphere (Table S5).

We next used LMMs to explore the most important driver of microbial alpha diversity. The analysis revealed that the plant compartment was the main factor influencing the alpha diversity of both bacterial and fungal communities based on Shannon diversity indices (*P* < 0.0001, Table S6). The effect of FWD on the alpha diversity was significant for both fungal (*P* = 0.00172) and bacterial (*P* = 0.023, Table S6) communities. Fungal alpha diversity was significantly lower in the upper stem epidermis, bottom stem epidermis, root endosphere, and rhizosphere soil under FWD than those in the healthy plants (*P* < 0.05, Fig. 1f). The sampling site had a prominent effect on the alpha diversity of bacterial communities (*P* = 0.006); however, it had no significant effect on the alpha diversity of fungal communities (*P* = 0.831, Table S6). In addition, the alpha diversity of fungal community in the fruit was not significantly different from that in the bulk soil in terms of Shannon diversity index and Chao1 richness index (*P* > 0.05, Fig. S5b and d).

**Fig. 2:**
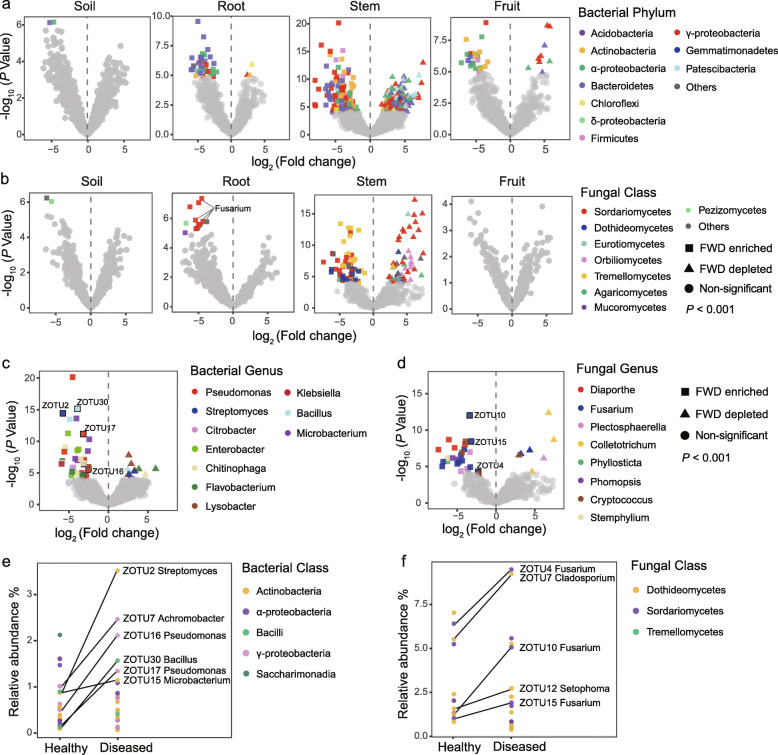
Volcano plot illustrating the enrichment and depletion patterns of bacterial and fungal microbiomes in the diseased organs compared with the healthy organs. **a** Effect of FWD on the abundance of bacterial ZOTUs (relative abundance > 0.1%, 2549 in total). The symbols correspond to FWD-enriched (square) and FWD-depleted (triangle) ZOTUs. **b** Effect of FWD on the abundance of fungal ZOTUs (relative abundance > 0.1%, 1030 in total). Note the functional guild information is presented in Fig. S6b. **c** 0.001).Relative abundance of potential beneficial bacteria is significantly increased in the diseased plants compared with the healthy plants (*P *< 0.001). The enrichment and depletion patterns of potential beneficial bacteria in the diseased root, stem, and fruit organs compared with healthy organs are presented in Fig. S6c. **d** (< 0.00Relative abundance of plant pathogenic fungi is significantly increased in the diseased plants compared with the healthy plants (*P* < 0.001).Taxonomic information for the top enriched and depleted taxa are provided in Table S8 and Table S9. **e** Several bacterial ZOTUs enriched in the diseased plants were also identified as the core bacterial taxa in both healthy and diseased plants **f** Several fungal ZOTUs enriched in the diseased plants were also identified as the core fungal taxa in both healthy and diseased plants

We identified 25 core bacterial taxa and 12 core fungal taxa in healthy plants, and 23 core bacterial taxa and 16 core fungal taxa in diseased plants (Additional file 2). Among these core taxa, 20 bacterial taxa and 12 fungal taxa were present in both healthy and diseased plants. Regarding the compositional variation, LMM analysis indicated that FWD had a significant effect on the relative abundance of class *Tremellomycetes* (*P* < 0.05), which belongs to saprotroph (Yeast) functional guild, but not on any bacterial phyla (Fig. 2b, Fig. S5h, Fig. S6a and b, and Table S7). Differential abundance analysis also indicated an increased abundance of *Tremellomycetes* in diseased plant stems at both sampling sites (Fig. S9 and Fig. S10b). Taxa enrichment and depletion in the diseased plants were more pronounced in the stem epidermis than in the xylem at both sampling sites, when compared with the healthy plant samples from the respective compartments (*P* < 0.001, Fig. S7–S9). The relative abundance of several potential pathogenic fungi from the genera *Diaporthe*, *Fusarium*,  *Phomopsis*, *Plectosphaerella*, *Stemphylium*, and *Cryptococcus* was also significantly higher in the diseased plant root and stem than in the healthy plant (*P* < 0.001, Fig. 2b and d). Three *Fusarium* ZOTUs (ZOTU4, ZOTU10, and ZOTU15) that were significantly enriched in the diseased plants were also identified as the core fungal taxa in both healthy and diseased plants (Fig. 2d and f, Additional file 2). However, several potential beneficial bacteria from the genera *Pseudomonas*, *Streptomyces*, *Klebsiella*, *Enterobacter*, *Microbacterium*, *Bacillus*, *Chitinophaga*, and *Citrobacter* were significantly enriched in the diseased plants (*P* < 0.001, Fig. 2b a and c, and Fig. S6c). For each site, the top 3 potential beneficial bacteria enriched in the diseased plant belonged to *Streptomyces*, *Microbacterium*, and *Pseudomonas* at Guiyang and *Bacillus*, *Bacillus*, and *Pseudomonas* at Huishui (*P* < 0.001, Fig. S10c). In addition, some potential beneficial bacteria that were significantly enriched in diseased plants, including *Streptomyces* (ZOTU2), *Pseudomonas* (ZOTU16), *Pseudomonas* (ZOTU17), and *Bacillus* (ZOTU30) were also identified as the core bacterial taxa in both healthy and diseased plants (Fig. 2c and e, Additional file 2).

### FWD affects pepper microbiome co-occurrence networks

**Fig. 3:**
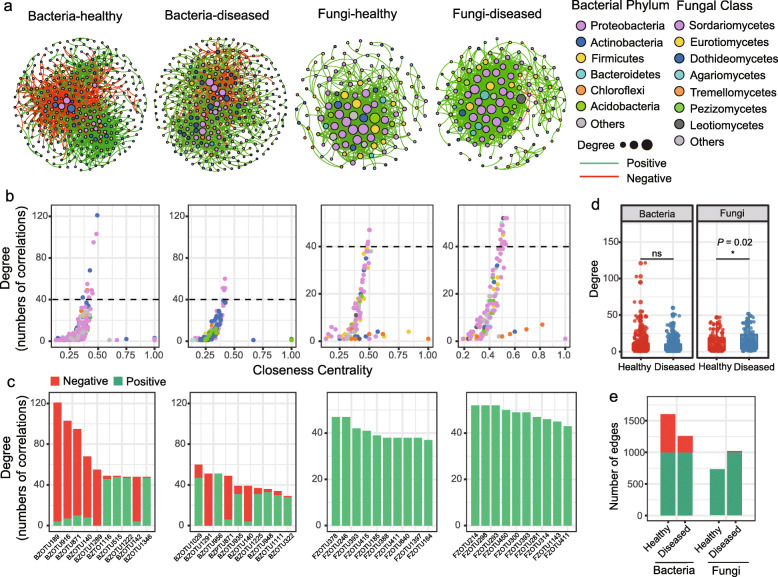
Intra-kingdom co-occurrence networks. **a** Intra-kingdom co-occurrence networks showing a higher number of nodes and edges in bacterial networks than those in fungal networks. The nodes are colored according to bacterial phylum and fungal class. Node size indicates the degree of connection. Edge color represents positive (green) and negative (red) correlations. **b** Comparison of node-level topological features in Fig. 3a (degree and closeness centrality) demonstrating the high degree and closeness centrality for the hub taxa. Taxonomic information for hub taxa is presented in Table S12. **c** Degree and interaction type of the top 10 hub nodes in four networks, showing a higher number of negative correlations in bacterial networks than in fungal networks. The degree (**d**) and edges (**e**) of bacterial and fungal taxa showing the higher complexity of the healthy bacterial network than that of the diseased bacterial network, with the opposite pattern showed in the fungal networks. The significance of difference was determined by nonparametric Kruskal–Wallis test

To investigate how FWD affects the pepper microbiome co-occurrence patterns, we analyzed the bacterial–bacterial and fungal–fungal intra-kingdom networks, as well as the bacterial–fungal interkingdom networks. Based on intra-kingdom network analysis, we recorded a higher proportion of negative edges and modularity in the bacterial networks (proportion of negative edges/modularity: 37.8%/0.464 in healthy and 19.9%/0.501 in FWD) than in the fungal networks (proportion of negative edges/modularity: 0%/0.269 in healthy and 1%/0.317 in FWD; Table S10). We also recorded a higher number of nodes and edges in the bacterial networks than in the fungal networks (Fig. 3a, Table S10). Further, the edges of top 10 hub nodes with high degree and closeness centrality values in the bacterial networks were primarily negative with other nodes, particularly in the healthy network (Fig. 3b and c). By contrast, most edges of the fungal networks were primarily positive (Fig. 3b and c). In addition, the bacterial network in healthy plants was more complex (based on the number of nodes and edges) than that in diseased plants; however, a contrasting pattern was observed for the fungal networks (Fig. 3b, d, and e, and Table S10), especially at the Huishui site (Fig. S11d).

**Fig. 4:**
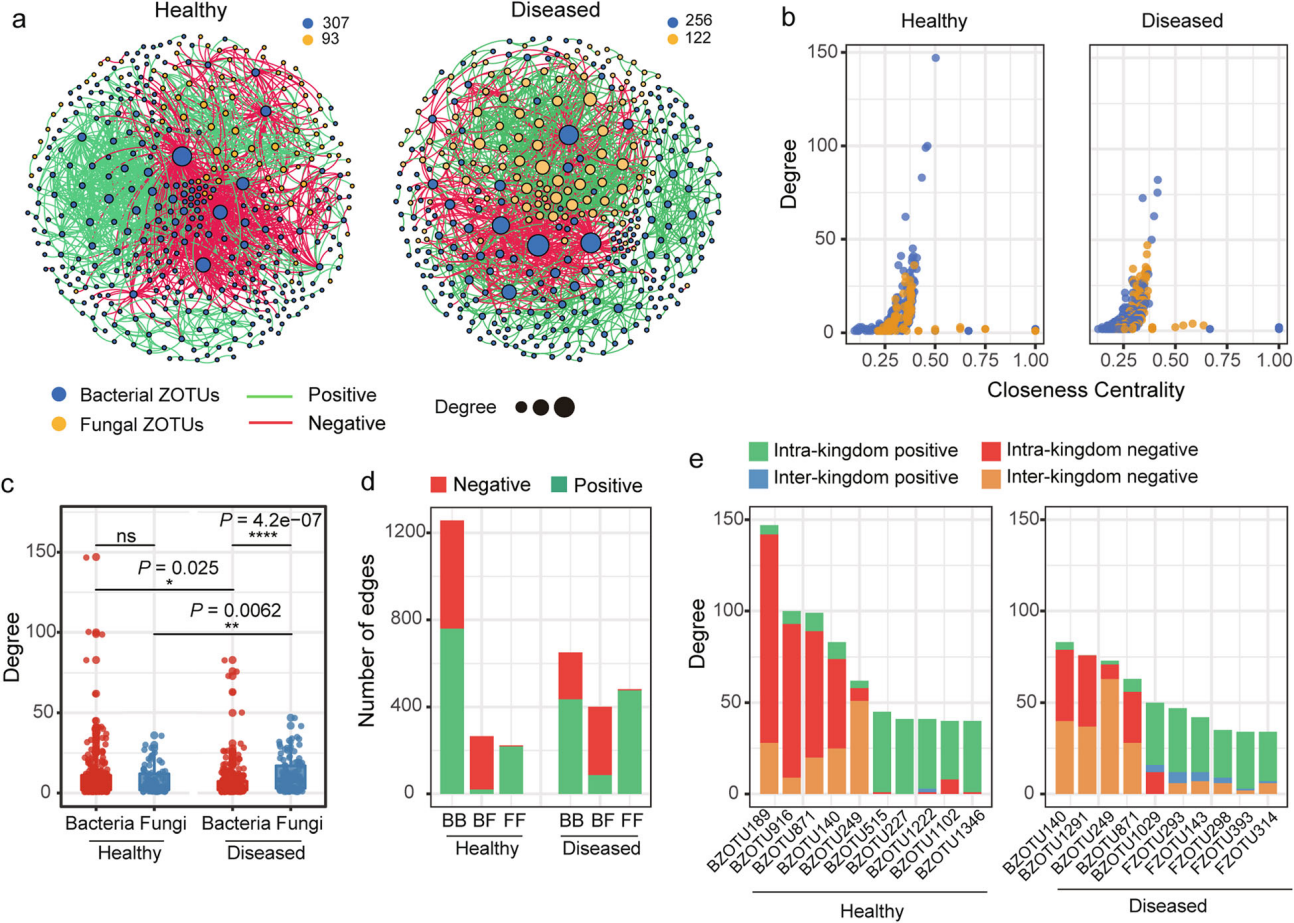
Interkingdom co-occurrence networks. **a** Networks contained both bacterial and fungal taxa showing a higher number of fungal taxa (orange) and a lower number of bacterial taxa (blue) in the diseased network than those in the healthy network. The networks in the soil, root, stem (upper, middle, and bottom), and fruit are presented in Fig. S12. **b** Comparison of node-level topological features in Fig. 4a (degree and closeness centrality) demonstrating the high degree and closeness centrality values for the hub taxa. Taxonomic information of the hub taxa is presented in Table S12. **c** Degree values of bacterial and fungal taxa in healthy and diseased networks. The significance of difference was determined by nonparametric Kruskal–Wallis test. **d** Number of bacterial–bacterial (BB), bacterial–fungal (BF), and fungal–fungal (FF) correlations in the healthy and diseased networks. Green and red colors of the edges and column indicate positive and negative correlations, respectively. **e** Degree and interaction type of the top 10 hub nodes in healthy (left) and diseased (right) networks. “Intra-kingdom correlation” refers to BB or FF, and “interkingdom correlation” refers to BF

The interkingdom co-occurrence networks further indicated that FWD destabilized the network and increased the intra-kingdom correlations among fungal taxa. The proportion of negative edges and modularity were higher in the healthy networks (proportion of negative edges/modularity: 42.8%/0.535) than in the diseased networks (proportion of negative edges/modularity: 34.9%/0.524; Table S10). The number of nodes and edges of fungal taxa was higher in the diseased network than in the healthy network, while an opposite pattern was observed among the bacterial taxa (Fig. 4a–c, Fig. S11e, and Table S10-S11). The BF (bacterial-fungal) interkingdom correlations were primarily negative (92.1% in healthy network and 78.3% in diseased  network), whereas positive correlations dominated the intra-kingdom correlations (60% BB and 98% FF in healthy plants network, and 66% BB and 99% FF in diseased network) (Fig. 4d). The top 10 hub taxa were bacterial in the healthy network, while fungal taxa accounted for half of the top 10 hubs in the diseased plant network (Fig. 4e Tableand Table S12). Similar patterns were apparent in most single-compartment networks (Fig. S12). Furthermore, several bacterial taxa, such as *Microbacterium*, *Streptomyces*, and *Pantoea*, enriched in the diseased plants were also identified as the top hub taxa in the networks (Table S8, Table S12, and Additional file 2).

### FWD affects pepper microbiome function

**Fig. 5:**
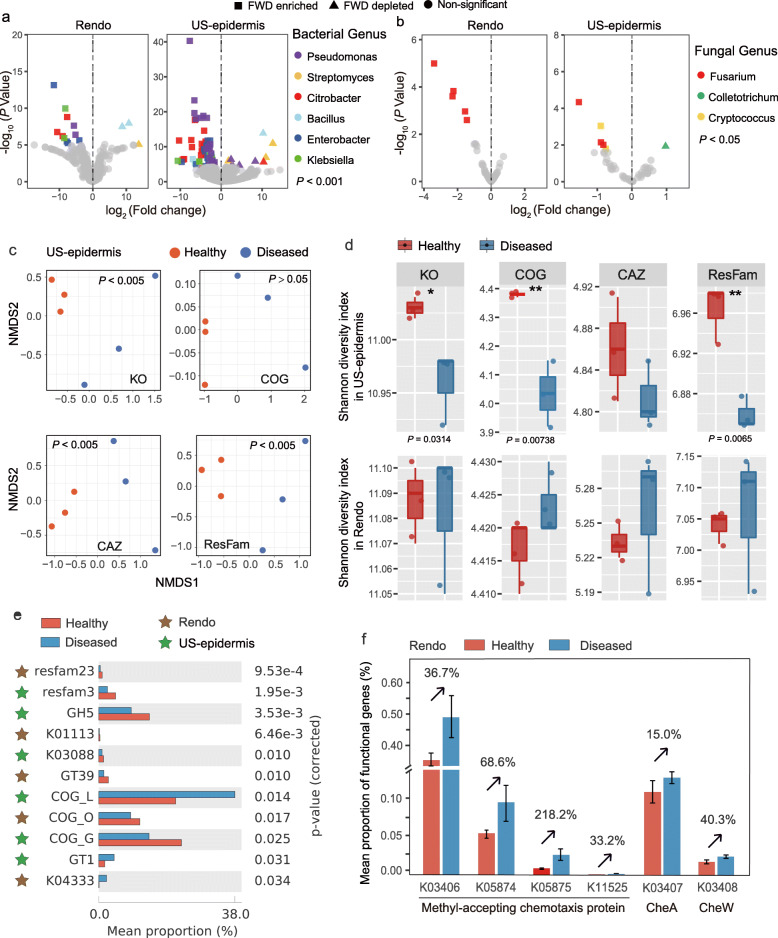
Microbiome functional diversity and differential abundance of functional genes/modules between the healthy and diseased plants based on KO, COG, CAZ, and ResFam functional profiles. **a** Enrichment and depletion of potential beneficial bacteria in the diseased plants compared with the healthy plants, as determined by metagenomic data analysis. "Rendo" represents root endosphere and "US-epidermis" represents upper stem epidermis. **b** Enrichment and depletion of potential pathogenic fungi in the diseased plants compared with the healthy plants, as determined by metagenomic data analysis. **c** NMDS ordinations of functional genes based on Bray–Curtis distance matrices of KO, CAZ, and ResFam functional genes showing the diseased upper stem epidermis microbiome significantly differed from that of the healthy plant. No such significant differences were apparent in the root endosphere microbiome (shown in Fig. S14a). **d** FWD significantly decreased the functional diversity of KO (*P* = 0.0314), COG (*P* = 0.0074), and Resfam (*P* = 0.0065) profiles in the upper stem epidermis microbiome, but showed no significant effect on the root endosphere microbiome (*P* > 0.05). **e** Differential abundance analysis of microbiome functional genes between the healthy (red) and diseased (blue) plants. **f** Relative abundance of microbiome functional genes involved in methyl-accepting chemotaxis proteins and their downstream targets in the root endosphere

We used metagenomic sequencing approach to explore the functional shift in the pepper-associated microbiomes that potentially induced by FWD. Since the microbiomes on upper stem epidermis and root endosphere present stronger responses to FWD than other compartments, we selected these two compartments from Huishui site for metagenomic sequencing. The metagenomic sequencing data were assigned to 6296 bacterial species and 57 fungal species. Differential abundance analysis of community composition revealed that several potential beneficial bacteria, such as *Enterobacter*, *Klebsiella*, *Citrobacter*, and *Pseudomonas* were significantly enriched in the root endosphere and upper stem epidermis compartments of the diseased plants (*P* < 0.001, Fig. 5a) when compared with the healthy plants. Several potential pathogenic fungi, such as *Fusarium* and *Cryptococcus*, were more abundant in the diseased plants than in the healthy plants (*P* < 0.05, Fig. 5b). These observations were consistent with the amplicon sequencing data (Fig. 2c and d).

Metagenomic analysis indicated that the functional composition (i.e., NMDS ordinations of KO, CAZ, and ResFam) of the diseased upper stem epidermis microbiome differed significantly from that of the healthy plant (*P* < 0.05, Fig. 5c), but not in the root endosphere microbiome (Fig. S14a). While FWD was linked to a decrease in the microbiome functional diversity of KO (*P* = 0.0314), COG (*P* = 0.0074), and Resfam (*P* = 0.0065) profiles of the upper stem epidermis, we did not observe significant changes on functional diversity in the root endosphere microbiome (Fig. 5d).

To determine how FWD affects the microbiome functional properties, we performed differential abundance analysis. The numbers of specifically enriched or depleted microbiome functional traits in the diseased upper stem epidermis were higher than those in the root endosphere (with healthy plants used as a baseline) (Table S13). Compared with the healthy plant, *phoD* alkaline phosphatase gene (K01113) and *mprF* peptide antibiotic resistance gene were depleted in the microbiome of the diseased root endosphere, while vancomycin resistance gene clusters were depleted in the microbiome of the diseased upper stem epidermis (*P* < 0.05, Fig. 5e, Fig. S14c and f, and Table S14). Further, a functional gene *csgD* related to LuxR family transcriptional regulator (K04333) was enriched in the microbiome of the diseased root endosphere;modules involved in UDP-glucuronosyltransferase (GT1) and replication, recombination, and repair (COG_L) were enriched in the microbiome of the diseased upper stem epidermis (*P* < 0.05, Fig. 5e, Fig. S14c–e, and Table S14). In addition, several functional genes involved in plant-microbiome signaling pathways (according to KO profile) were more abundant in the microbiome of the diseased root endosphere than in the healthy. For instance, the relative abundance of genes associated with methyl-accepting chemotaxis proteins (MCPs; K03406, K05874, K05875, and K11525) was increased by 33.2–218.2% in the microbiome of the diseased root endosphere, compared with the healthy plant (Fig. 5f). The relative abundance of the functional genes associated with the downstream of MCPs, such as histidine kinase CheA (K03407) and purine-binding chemotaxis protein CheW (K03408), also increased by 15.0–40.3% in the microbiome of the diseased root endosphere, compared with the healthy plant (Fig. 5f).

## Discussion

In this study, we sought to investigate the effect of FWD on chili pepper microbiomes using amplicons and metagenomic approaches. By profiling both bacterial and fungal communities in twelve below- and aboveground compartments of healthy and FWD pepper plants, we reveal that fungal networks are less stable and their communities are more sensitive to FWD than the bacterial communities. FWD has a stronger impact on the microbiome assembly of vegetative organs than on those of reproductive organs, with the strongest effects on the upper stem epidermis and root endosphere. Metagenomic sequencing data from these two compartments further suggested that several functional genes involved in detoxification, biofilm formation, and plant–microbiome signaling pathways (i.e., chemotaxis) were significantly enriched in the FWD plants. Moreover, our work provides evidence that other organs of pepper plants besides the root, such as stem and fruit, can also recruit potential beneficial bacteria to the FWD plants. Through this work, we provide evidence that FWD not only changes the diversity, assembly, and network of microbial communities, but also impacts their ecological functions. Below, we discuss how these findings have advanced our understanding of disease-induced changes in plant microbiome assembly, co-occurrence patterns and functions.

### FWD affects less on the microbiome of reproductive organs than vegetative organs

Uncovering how the host plant and its associated microbiomes respond to plant disease is of great importance to advance the co-evolutionary theory of plant-microbiome interactions [87]. Our study demonstrated that FWD affects the bacterial and fungal communities in the reproductive organ (fruit) to a lesser extent than those in vegetative organs (root or stem). Changes in the fungal community were associated with co-infection with other potential fungal pathogens in the root and stem, but not in the fruit. Thus, the less-pronounced effect of FWD on the fruit, relative to that on the root and stem, may represent a life history tradeoff strategy of a plant to ensure survival of the next generation (fruit and seed) rather than investing in the contemporary diseased individual. Secondary metabolites, such as capsaicinoids, may protect the chili fruit and seed from fungal pathogens [88].

The strongest effect of FWD on microbial communities was in the upper stem epidermis compared with other soil and plant compartments. FWD-induced changes in plant physiological characteristics, such as water relations [89], could strongly affect the aboveground parts of the plant. For the stem, the effect of FWD on both bacterial and fungal communities in epidermis compartments was more pronounced than that on those in the xylem compartments. The epidermis is a more favorable niche for microbes than the xylem in terms of accessibility of organic nutrients (such as small sugars) [90, 91]. For the root, a pronounced effect of FWD on the bacterial and fungal communities was observed in the endosphere than in the episphere, for which the epidermis and xylem were not considered separately in the current study. The episphere was supposed to be an important interface between the host and the environment, and the root episphere microbiomes were determined by both host selection and soil characteristics [13, 14]. Since fungi are the important consumers of belowground inputs of plant-derived carbon [50–52], the mycobiome in the root endosphere could respond strongly to FWD.

The microbial communities in the diseased plants were more variable than those in the healthy plants in most compartments. This is contrary to the expectation, based on homogeneous selection [92], that the same environmental selection pressure often leads to similar community structures. Plant-associated microbiomes were shaped by multiple host and environmental factors, such as plant compartment, host genetics, and edaphic factors. A recent study indicated that host selection (i.e., compartment niche and host species) has a greater determining effect on shaping the plant microbiome than the environmental factors [14]. The pronounced effect of the host compartment observed herein for pepper has been also observed in sorghum [93] and *Populus* [16, 94]. The current study provides additional evidence in support of the niche occupation theory of plant microbiome assembly [37, 94] under both healthy and diseased conditions. Having observed a predominant effect of the host compartment on microbial community composition, we propose that disease may lessen the plant effect and, thereby, potentiate community dissimilarity in the diseased plant.

### Fungal communities are more sensitive to FWD than bacterial communities

Cooperative and competitive interactions among microbial species and network modularity can influence the community stability [40, 95]. In this study, bacterial networks and their hub taxa in both healthy and diseased plants were characterized by a higher proportion of negative correlations than those in the fungal networks. Mutually negative interactions, indicating ecological competition, can improve microbiome stability by dampening the destabilizing effects of cooperation [40]. The host may benefit from microbial competition, which results in improved resistance to external stress [53]. In contrast to bacterial communities, the fungal communities were more affected by FWD, probably due to enhanced positive intra-kingdom correlations among fungal taxa observed in FWD networks as compared with the healthy networks. Also, lower modularity in fungal network may exacerbate the destabilizing effect due to the higher prevalence of cross-module correlations among taxa [39, 41]. These findings indicate that fungal community was more sensitive to FWD than bacterial communities as demonstrated by its lower network stability. A previous study reported that soil bacterial networks were less stable under drought stress than fungal networks [17]. Since our samples were plant-associated compartments and the external stress is biotic, these could account for the contrasting results.

Our results indicated that sampling site had a higher impact on the bacterial community than on the fungal community. The sampling site effect represented the interaction effect of site-dependent environmental characteristics (e.g., climate and soil type) and the cultivar (host genotype) at each site, which may co-influence the microbiome composition. Bacteria and fungi differ in body size [96, 97], diversity, metabolic activity [98], dispersal potential [99], and the interaction with host or other microbes, which may affect species sorting and the community assembly process.

Our data indicated that FWD decreased the complexity of bacterial networks but increased the complexity of fungal networks. The contrasting pattern between the bacterial and fungal networks parallels recent observation based on soil macroecological patterns of *Fusarium* wilt [100]. Previous study has revealed the importance of the network complexity [53] and hub taxa [101, 102] in supporting ecosystem functions. The fungal connectivity, mainly belonging to intra-kingdom cooperative interactions, increased in the diseased plants, thus inducing the ecological importance of fungal taxa. In addition, we found the cooperative correlations dominated within each microbial kingdom but the competitive correlations dominated between bacteria and fungi, which may be explained by the fact that the bacteria and fungi normally compete for plant-derived substrates [52].

### Disease-induced changes in microbiome composition and functions

Deciphering the keystone taxa (e.g., biomarker taxa, core taxa, and network hubs), and their correlations with the host plant and pathogens, is critical for harnessing the plant microbiome to enhance plant growth and health [4, 12]. Several potential beneficial bacteria, such as *Pseudomonas*, *Streptomyces*, and *Bacillus*, were enriched in diseased plants in the current study, which were also identified as the core taxa (i.e., present in all samples) in plant microbiomes. Previous studies have revealed that many members of the *Pseudomonas*, *Streptomyces*, and *Bacillus* genera colonize different plant compartments (e.g., phyllosphere and rhizosphere) and play a vital role in modulating host performance, especially in plant pathogen suppression [4, 54, 87, 103, 104]. For example, *Streptomyces* is well known for excreting antibiotic compounds and can protect plants from pathogens [105–107]. *Pseudomonas* and *Bacillus* are the two most dominant taxa of plant-beneficial bacteria, and some representatives of these two genera can coexist and cooperate with each other [21]. Our results indicated that the host plant may selectively regulate the community abundance of some core taxa under pathogen stress. Further, several bacterial taxa, such as *Microbacterium*, *Streptomyces*, and *Pantoea*, were enriched in diseased plants and were also identified as hub taxa in the co-occurrence networks. Hub taxa hold key topological positions within the network and may be deployed to organize favorable plant microbiomes [12]. For instance, a study on *Arabidopsis thaliana* suggested that the host plant selectively impacts its associated microbiomes and microbe-microbe interactions by modulating the hub taxa *Albugo laibachii* and *Dioszegia* spp. in the phyllosphere [15]. The overlap between the biomarker taxa, core taxa, and network hubs suggests that some bacterial taxa recruited by the diseased plants may act as keystone taxa for plant microbiomes and ensure the survival of the next generation.

The current study provides evidence on the critical role of bacterial taxa in the “cry for help” strategy of the host plant, in which the plant actively involves its microbial partners to maximize its or its offspring survival and growth under external stress. This is a survival strategy conserved across the plant kingdom [18, 25, 87]. For example, a study of sugar beet *Rhizoctonia* damping-off disease indicated that members of the *Chitinophagaceae* and *Flavobacteriaceae* become enriched within the plant endosphere upon pathogen invasion and that reconstruction of a synthetic community of *Flavobacterium* and *Chitinophaga* consistently suppresses fungal root disease [11]. Several recent studies also suggested that the aboveground pathogen infection induces an assemblage of a plant-beneficial bacterial consortium in the root microbiome [23, 26]. Berendsen et al. [23] reported that *A. thaliana* specifically promotes three bacterial taxa (*Stenotrophomonas* sp., *Xanthomonas* sp., and *Microbacterium* sp.) in the rhizosphere upon foliar infection with *Hyaloperonospora arabidopsidis*, and together these three bacteria will induce systemic resistance against pathogen and promote growth of the plant. Similarly, based on the pepper data presented in the current study, an infection with a soil-borne pathogen (e.g., FWD) has driven the recruitment of beneficial microbes to the aboveground parts of the host plant. Intriguingly, Liu et al. [22] provided evidence for the recruitment of beneficial microbes to the wheat rhizosphere and root endosphere to suppress the soil-borne pathogen *Fusarium pseudograminearum*. The study also showed that the beneficial microbe *Stenotrophomonas rhizophila* could boost plant defenses in the aboveground parts when the pathogen was present.

Metagenomic analyses indicated that microbiome functional genes involved in detoxification, chemotaxis, and biofilm formation were enriched in the diseased plant compared with the healthy plant. UDP-glucuronosyltransferases were enriched in the microbiome from the upper stem epidermis of diseased pepper. UDP-glucuronosyltransferases encode a family of detoxifying enzymes [108–110] that may detoxify the toxic metabolites, such as fusaric acid, trichothecenes, fumonisins, and enniatins produced by *Fusarium* spp. [45, 111] or by other co-infected pathogenic fungi. *CsgD* LuxR family transcriptional regulator was enriched in the microbiome of diseased root endosphere. This is the master regulator of biofilm formation pathway, which could protect microbes from the adverse environmental conditions, thereby enhancing microbial survival [112–114]. Several genes encoding MCPs associated with plant-microbiome signaling pathways were enriched in the microbiome of diseased root endosphere. MCPs are the predominant chemoreceptors in motile bacteria that alter the activity of CheA histidine kinase and the bacterial swimming behavior upon detection of specific chemicals [115]. MCPs have been identified in typically beneficial bacteria, e.g., *Bacillus subtilis* [116] and *Pseudomonas* spp. [117, 118], which were also significantly enriched in diseased plant in the current study. Under stress conditions, such as pathogen invasion, a plant can attract distant beneficial microbes by actively releasing nonvolatile root exudates, such as amino acids, nucleotides, and long-chain organic acids [26], or by actively emitting blends of volatile organic compounds [24]. The findings of the current study suggest that the MCP gene enrichment in diseased plants may be related to the response of MCP-producing bacteria to plant-released signal molecules. These bacteria would use MCPs to detect specific concentrations of these molecules in the extracellular matrix, enabling directional accumulation of the bacteria to the plant. Although the taxonomic and functional analyses of healthy and diseased pepper microbiomes provide evidence for the plant “cry for help” strategy, culture-based experiments are required to verify the hypothesis. Specifically, the enriched potential beneficial bacteria should be isolated and their disease-suppressing effects tested in vivo. The putative plant signal molecules released under biotic stress are also worthy of further exploration.

Finally, metagenomic analyses revealed that FWD significantly decreased the functional diversity of KO, COG, and Resfam profiles of the microbiome of the upper stem epidermis. The functional diversity reduction could be largely caused by a drop in microbial diversity. A number of studies have demonstrated the importance of biodiversity for ecosystem function [119–122]. Similarly, our data showed that high microbiome diversity in healthy plant could ensure its better involvement in multiple ecosystem functions. Highly diverse microbiome communities tend to be more complex and possess greater functional redundancy and interkingdom associations [53]. By contrast, pathogen invasion could reduce the microbiome diversity and functional diversity as a result of disease-induced inhibition of plant photosynthesis [123] and change in water physiological characteristics [89]. In the current study, the relative abundance of alkaline phosphatase gene *phoD*, which is responsible for the recycling of organic phosphorus, was reduced in the diseased root endosphere, suggesting that the FWD affects plant phosphorus absorption [124]. Greater functional variation in the upper stem epidermis microbiome than that in the root endosphere microbiome may also reflect the density of microbes surrounding each plant organ, which is vastly greater in the root than in the stem [36].

## Conclusions

Based on the presented data, the host compartment exerts the strongest effect on the bacterial and fungal microbiome assembly, followed by FWD, and the sampling site. Fungal communities are more sensitive to FWD than bacterial communities, and fungal taxa play a more important role in the diseased co-occurrence interkingdom network than the healthy network. Microbiomes of the reproductive compartments are less affected by FWD than those of the vegetative compartments. The compartments of diseased pepper plant may recruit beneficial bacterial taxa that could provide protective functions to host plants. The current study siginificantly improves our understanding on microbiome assembly and function in both the below- and aboveground compartments of chili pepper under FWD and provids potential for manipulating the plant microbiome to promote plant health and sustainable agricultural production.

## Supplementary Information

**Additional file 1.Fig. S1** Pathogen isolation, identification, and pathogenicity test. **Fig. S2** Samples were divided into different compartments for preparing of DNA extraction.** Fig. S3** NMDS of bacterial communities in soil, root, stem (3 sections), and fruit. **Fig. S4** NMDS of fungal communities in soil, root, stem (3 sections), and fruit. **Fig. S5** Changes of alpha diversity indices and taxonomic composition of bacterial and fungal communities. **Fig. S6** Relative abundance of differentially abundant taxa between healthy and diseased plant. **Fig. S7** The volcano plots illustrating the enrichment and depletion patterns of the bacterial and fungal microbiomes in FWD plant compartments compared with the healthy. **Fig. S8** The volcano plots illustrating the enrichment and depletion patterns of the bacterial class in FWD plants compartments in Guiyang (top) and Huishui (bottom), when the healthy plants were used as a baseline. **Fig. S9** The volcano plots illustrating the enrichment and depletion patterns of the fungal phylum in FWD plants compartments in Guiyang (top) and Huishui (bottom), when the healthy plants were used as a baseline. **Fig. S10** The volcano plots illustrating the enrichment and depletion patterns of microbiome in FWD plants all compartments in Guiyang (left) and Huishui (right), when the healthy plants were used as a baseline. **Fig. S11** Intra- and interkingdom co-occurrence networks at Guiyang and Huishui. **Fig. S12** Interkingdom co-occurrence networks in soil, root, stem (3 sections), and fruit. **Fig. S13** Taxonomic composition and differentially abundant taxa of bacterial and fungal communities between healthy and diseased root endosphere and upper stem epidermis from metagenomic sequencing data. **Fig. S14** Changes of microbiome functional profiles between healthy and diseased root endosphere and upper stem epidermis. **Table S1**. Primers information used in this study. **Table S2**. PERMANOVA by adonis of all bacterial 16S and fungal ITS samples. **Table S3**. PERMANOVA by adonis of bacterial 16S conducted separately for each compartment. **Table S4**. PERMANOVA by adonis of fungal ITS conducted separately for each compartment. **Table S5**. Distance to centroid was calculated by analysis of beta-dispersion using Bray–Curtis dissimilarity. **Table S6**. Linear-mixed model (LMM) for alpha diversity indices. **Table S7**. Linear-mixed model for bacterial phylum and fungal class composition. **Table S8**. Differentially abundant analysis showing the enrichment and depletion patterns of bacterial microbiomes in diseased organs compared with healthy organs. **Table S9**. Differentially abundant analysis showing the enrichment and depletion patterns of fungal microbiomes in diseased organs compared with healthy organs. **Table S10**. Topology properties of the intra- and interkingdom networks. **Table S11**. The taxonomic composition of bacterial phylum and fungal class between healthy and diseased intra- and interkingdom networks. **Table S12**. The taxonomic position of top 10 hubs in intra- and interkingdom networks. **Table S13**. Numbers of enriched and depleted functions in diseased plant compared with the healthy plant. **Table S14**. Functional annotation of differentially abundant genes (top 20) between healthy and diseased plant calculated by LEfSe difference analysis.

**Additional file 2.** Additional data about the taxonomy information of core taxa for bacterial and fungal communities and the network hub taxa.

## Data Availability

The raw sequencing data have been deposited in the NCBI Sequence Read Archive (SRA) database under the accession number PRJNA667302 (16S), PRJNA667299 (ITS), and PRJNA667562 (metagenomic).
