# Supplementary material for: Disease-induced changes in plant microbiome assembly and functional adaptation
Source: Microbiome. 2021 Sep 15;9:187. doi: 10.1186/s40168-021-01138-2 (PMC8444440; doi:10.1186/s40168-021-01138-2)
Supplement: Supplementary file 2 — Additional file 1.Fig. S1 Pathogen isolation, identification, and pathogenicity test. Fig. S2 Samples were divided into different compartments for preparing of DNA extraction. Fig. S3 NMDS of bacterial communities in soil, root, stem (3 sections), and fruit. Fig. S4 NMDS of fungal communities in soil, root, stem (3 sections), and fruit. Fig. S5 Changes of alpha diversity indices and taxonomic composition of bacterial and fungal communities. Fig. S6 Relative abundance of differentially abundant taxa between healthy and diseased plant. Fig. S7 The volcano plots illustrating the enrichment and depletion patterns of the bacterial and fungal microbiomes in FWD plant compartments compared with the healthy. Fig. S8 The volcano plots illustrating the enrichment and depletion patterns of the bacterial class in FWD plants compartments in Guiyang (top) and Huishui (bottom), when the healthy plants were used as a baseline. Fig. S9 The volcano plots illustrating the enrichment and depletion patterns of the fungal phylum in FWD plants compartments in Guiyang (top) and Huishui (bottom), when the healthy plants were used as a baseline. Fig. S10 The volcano plots illustrating the enrichment and depletion patterns of microbiome in FWD plants all compartments in Guiyang (left) and Huishui (right), when the healthy plants were used as a baseline. Fig. S11 Intra- and interkingdom co-occurrence networks at Guiyang and Huishui. Fig. S12 Interkingdom co-occurrence networks in soil, root, stem (3 sections), and fruit. Fig. S13 Taxonomic composition and differentially abundant taxa of bacterial and fungal communities between healthy and diseased root endosphere and upper stem epidermis from metagenomic sequencing data. Fig. S14 Changes of microbiome functional profiles between healthy and diseased root endosphere and upper stem epidermis. Table S1. Primers information used in this study. Table S2. PERMANOVA by adonis of all bacterial 16S and fungal ITS samples. Table S3. PERMANOVA by adonis [file 40168_2021_1138_MOESM2_ESM.docx]

## Supplementary Information

**Supplementary figures**

**Fig. S1** Pathogen isolation, identification, and pathogenicity test. **a** Pathogen isolation. Pathogen isolated from the diseased pepper had clear disease symptoms (brown vascular bundle). The tissue of vascular bundle was placed into potato dextrose agar (PDA; Merck, Darmstadt, Germany) and incubated for 7 days in the dark at 25°C. Pure culture isolates were obtained by the single-spore technique. Figures in the upper right corner showing the conidiophores and phialides on aerial mycelium. **—** Scale bars = 10 μm. **b** Pathogenicity test. Pepper seedlings with wounded roots were submerged into a conidial suspension (10^5^ CFU/ml) 1 hour before transplanted, while control plants were dipped into sterile water. Wilt symptoms developed in all inoculated plants 7 to 14 days after inoculation, and infected plants developed the same symptoms as observed on the original plants from which the pathogen was isolated, while control treatment remained symptomless. The same pathogen was consistently reisolated from infected plants. All experiments were conducted with three replicates, and the pathogenicity test was conducted twice with the similar results. **c** The PhyML tree (conducted in: http://www.atgc-montpellier.fr/phyml/) of *Fusarium oxysporum* species complex inferred from the combined TEF and RPB2 sequence alignments. The tree is rooted with *F. foetens* (CBS 120665) and *F. udum* (CBS 177.31). ^T^ indicates Epi- and ex-type strains.


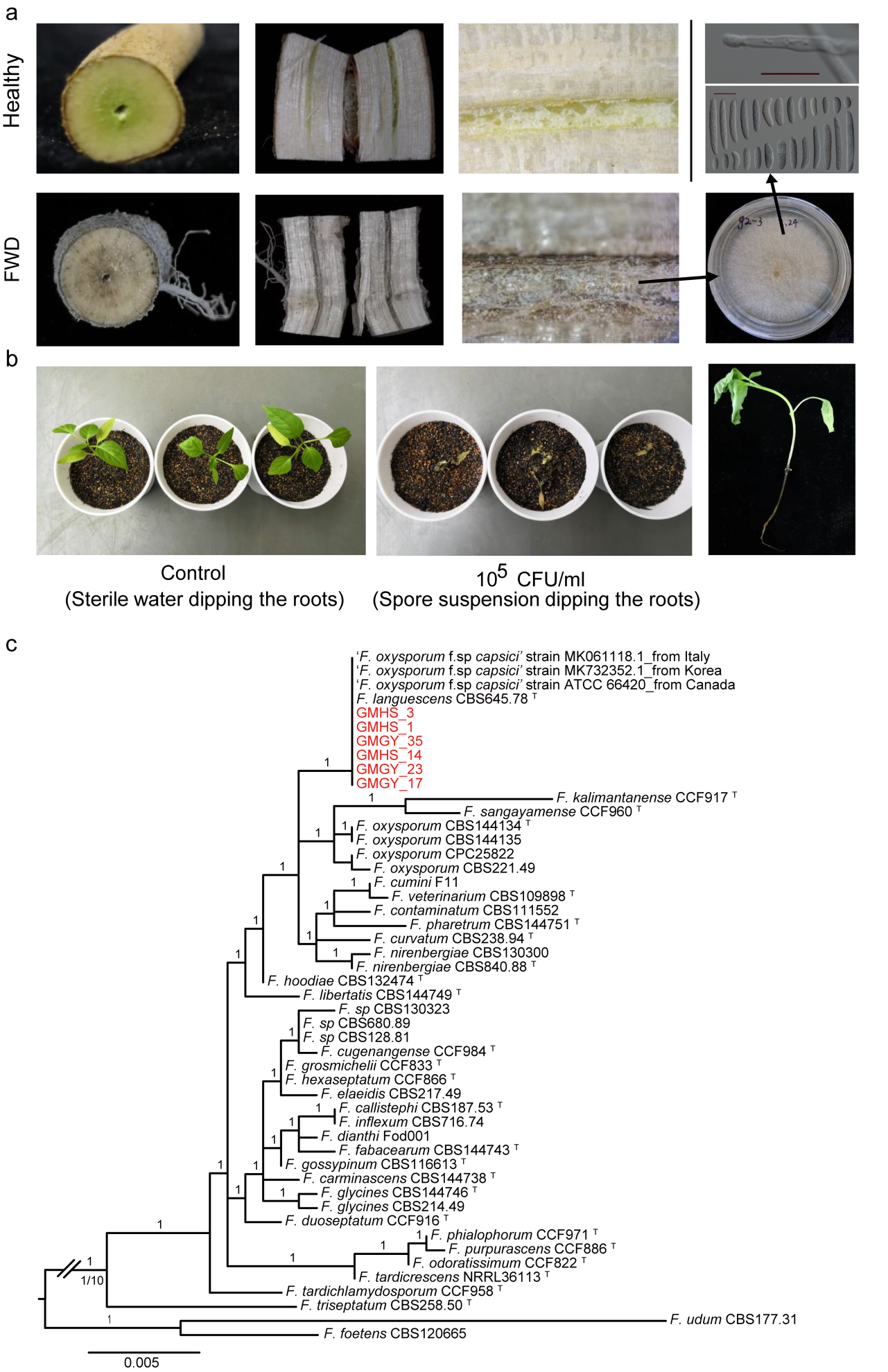


**Fig. S2** Samples were divided into different compartments for DNA extraction. Apart from the compartments shown in the figures, bulk soil and rhizosphere soil were also included. Above all, there were12 plant compartments in this study.


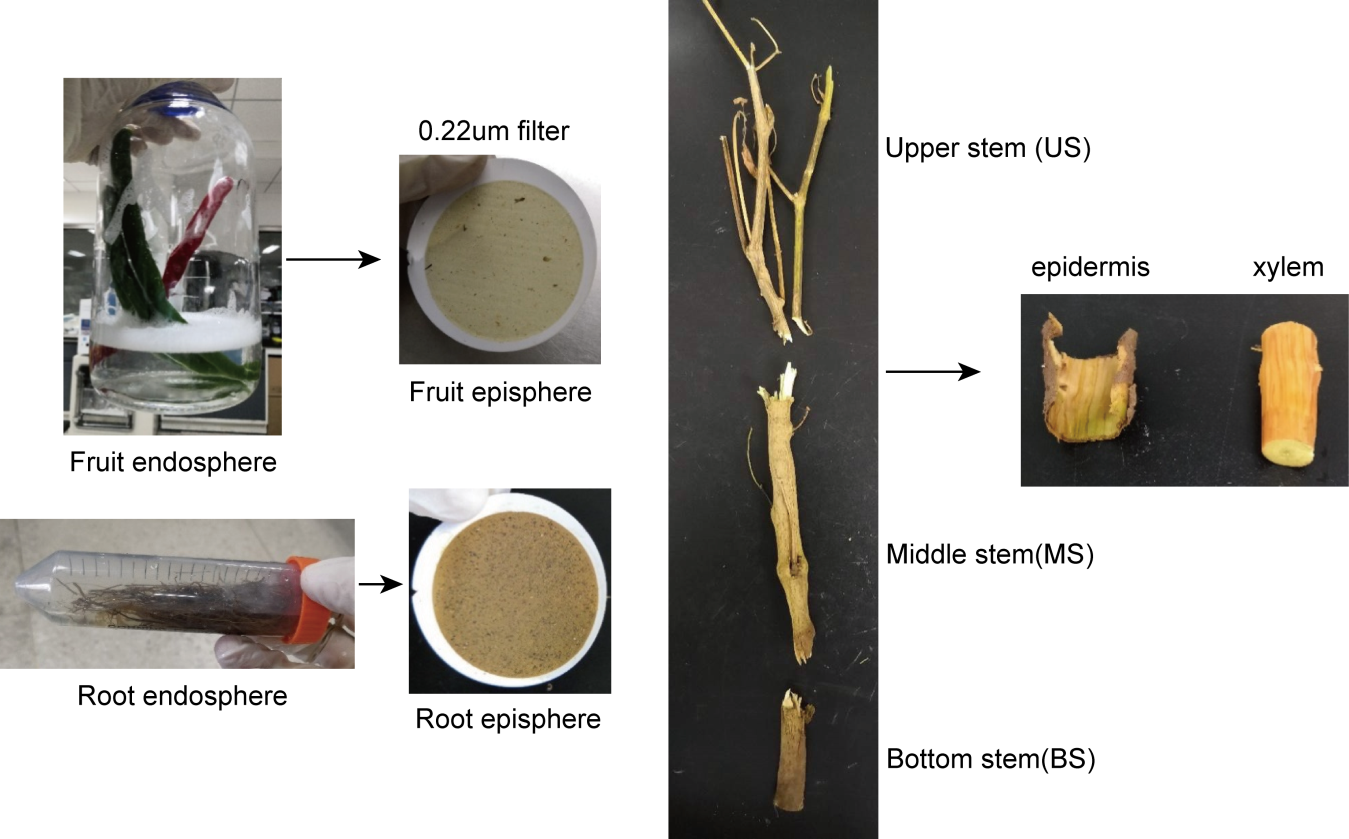


**Fig. S3** NMDS of bacterial communities in soil, root, stem (3 sections), and fruit. Different color represents different plant compartments. Solid and hollow represent two different sampling sites, and the different symbols correspond to healthy (triangle) and diseased (square) plant, respectively.


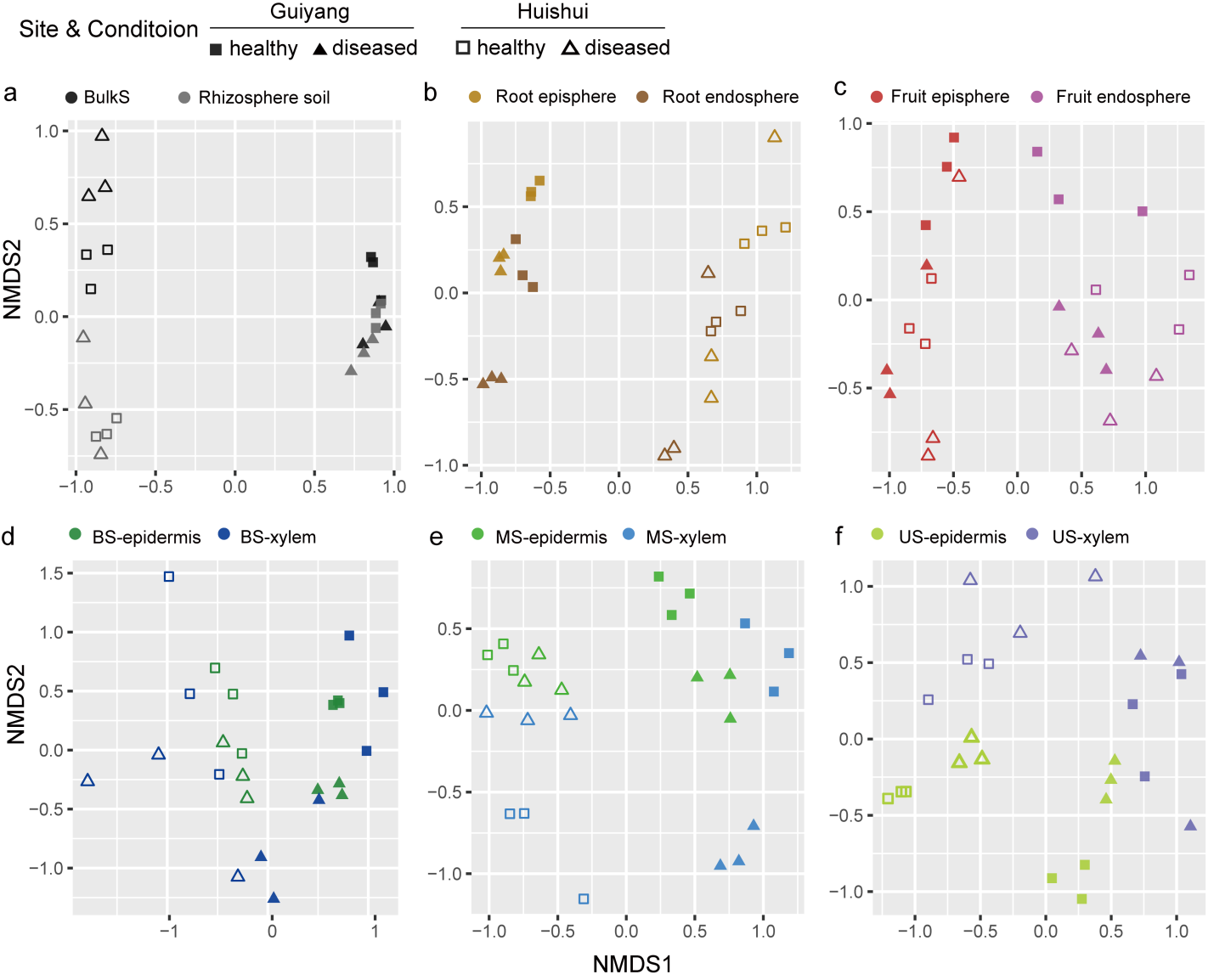


**Fig. S4** NMDS of fungal communities in soil, root, stem (3 sections), and fruit. Different color represents different compartments. Solid and hollow represent two different sampling sites, and different symbols correspond to healthy (triangle) and diseased (square) plant, respectively.


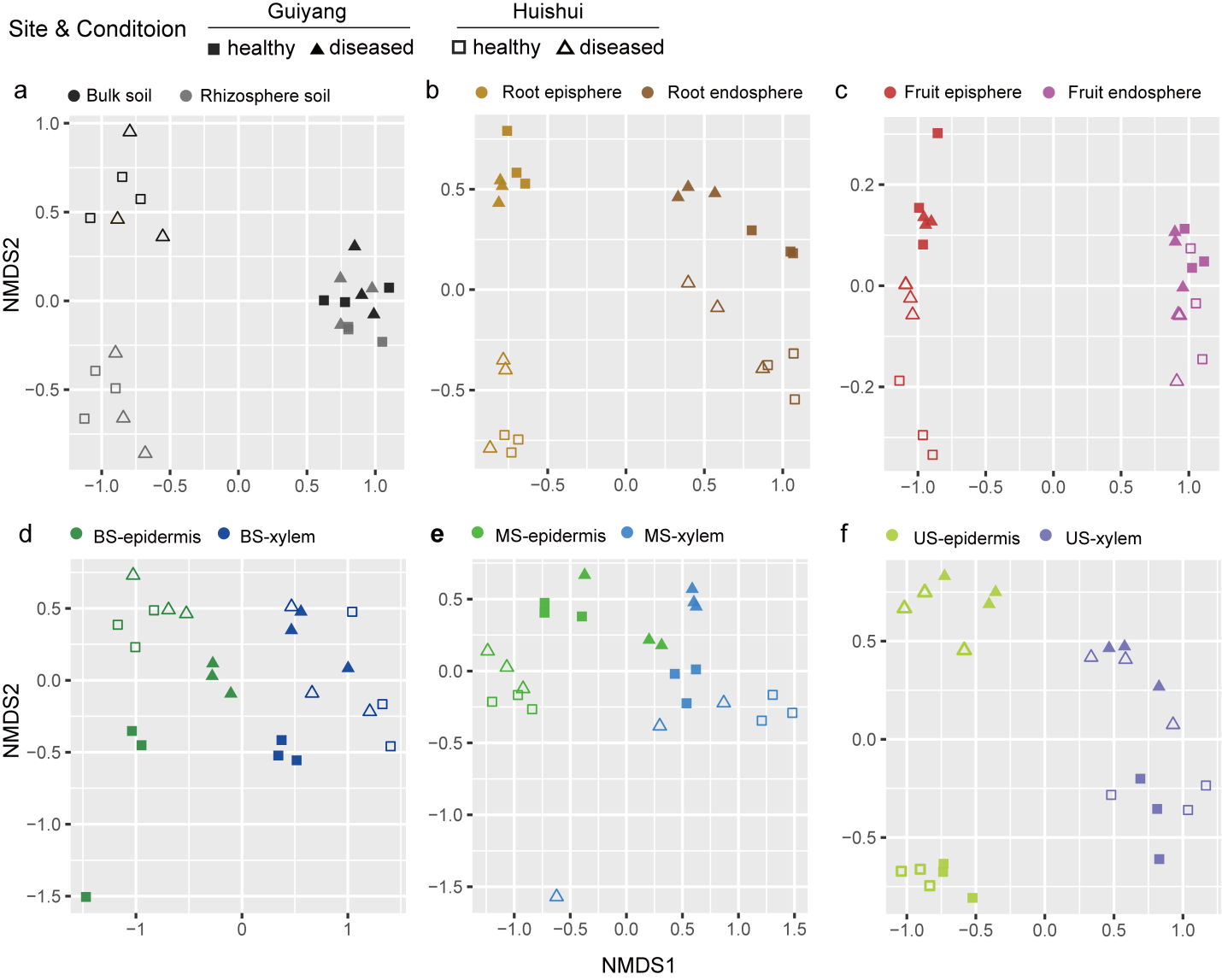


**Fig. S5** Changes in alpha diversity indices and taxonomic composition of bacterial and fungal communities. **a–b** Changes in Shannon diversity index of bacterial and fungal community in different compartments. **c–d** Changes in Chao1 richness index of bacterial and fungal community in different compartments. **e–f** Changes in Chao1 richness index of different compartments in healthy (red color) and diseased (blue color) plant bacterial and fungal community. **g–h** Relative abundance of bacterial phyla and fungal classes varied between healthy and diseased plant. Phyla or classes less than 1% of the total reads were grouped into “Low abundance”.

**
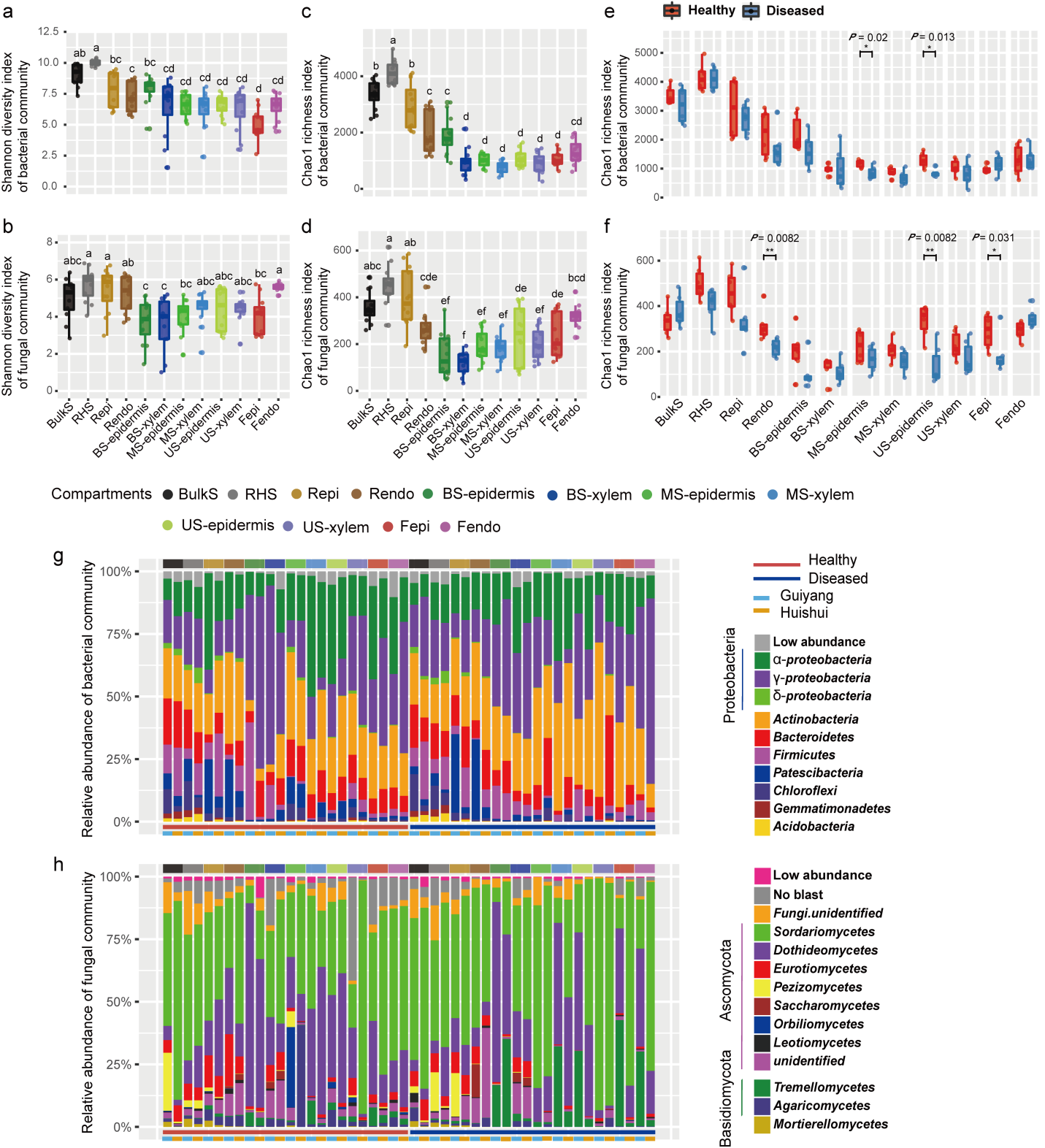
**

**Fig. S6** Relative abundance of differentially abundant taxa between healthy and diseased plant. **a** Relative abundance of *Tremellomycetes* between healthy (red color) and diseased (blue color) plant in different compartments. **b** Volcano plots illustrating the enrichment and depletion patterns of the fungal functional guild in diseased pepper stem, root, and fruit organ compared with the healthy. **c** Volcano plot illustrating the enrichment and depletion patterns of the potentially beneficial bacteria in diseased pepper stem, root, and fruit organ compared with the healthy. The symbols correspond to FWD-enriched (square) and -depleted (triangle) ZOTUs.


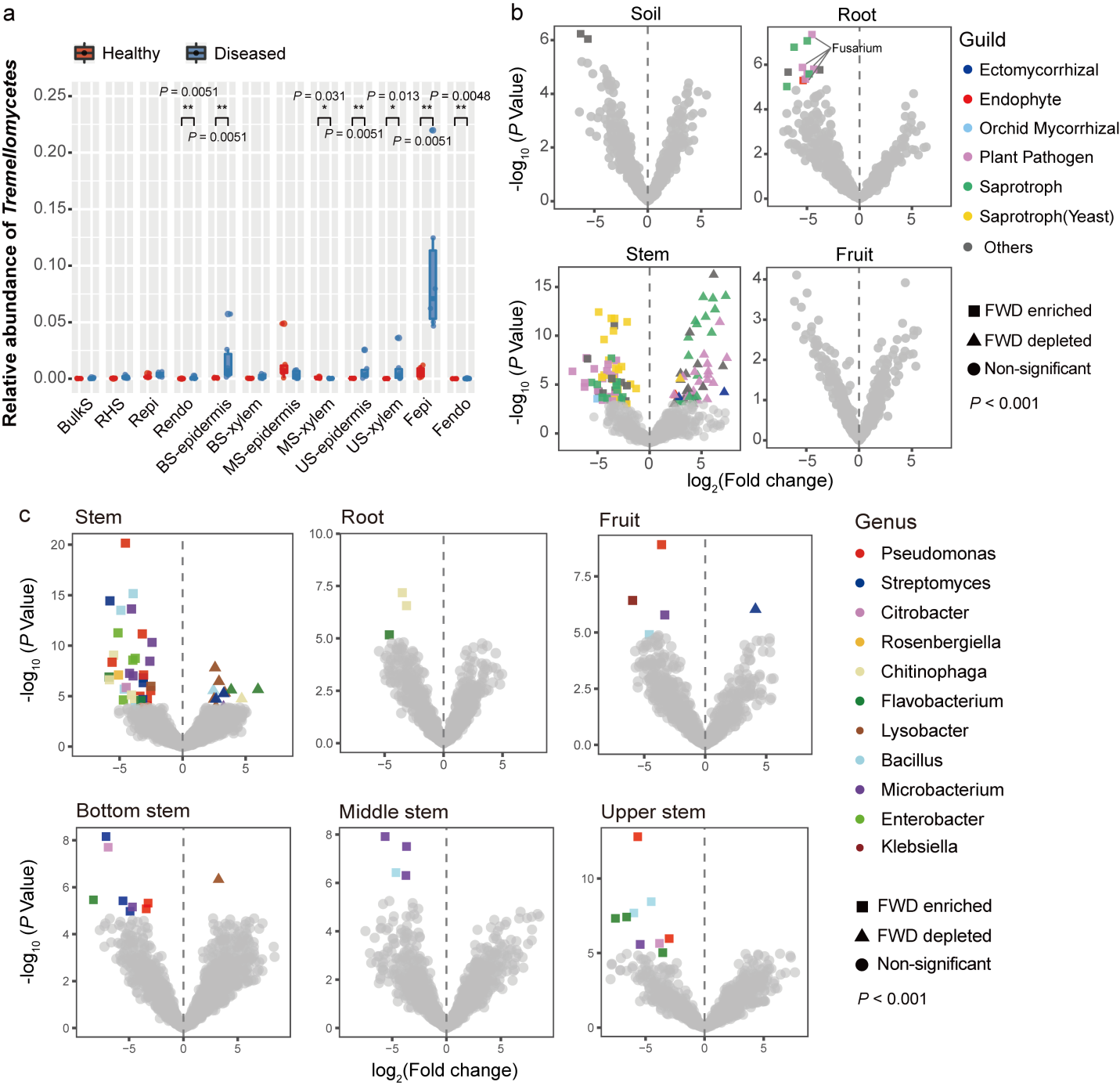


**
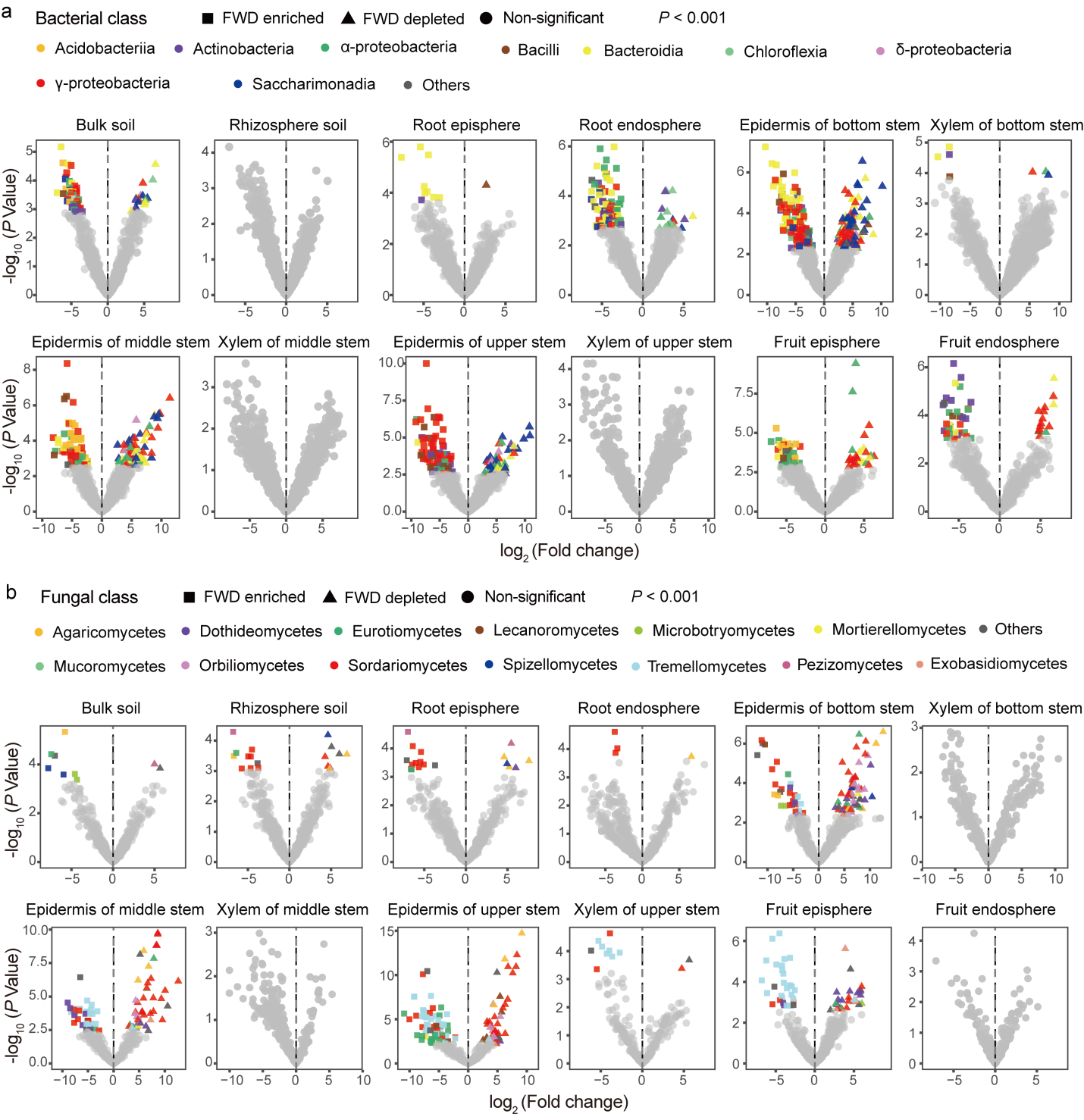
Fig. S7** The volcano plots illustrating the enrichment and depletion patterns of the bacterial and fungal microbiomes in FWD plant 12 compartments, when the healthy plants were used as a baseline. **a** Volcano plots showing FWD plants enriched and depleted bacterial class at 12 compartments. **b** Volcano plots showing FWD plants enriched and depleted fungal class at 12 compartments. The symbols correspond to FWD-enriched (square) and -depleted (triangle) ZOTUs.

**
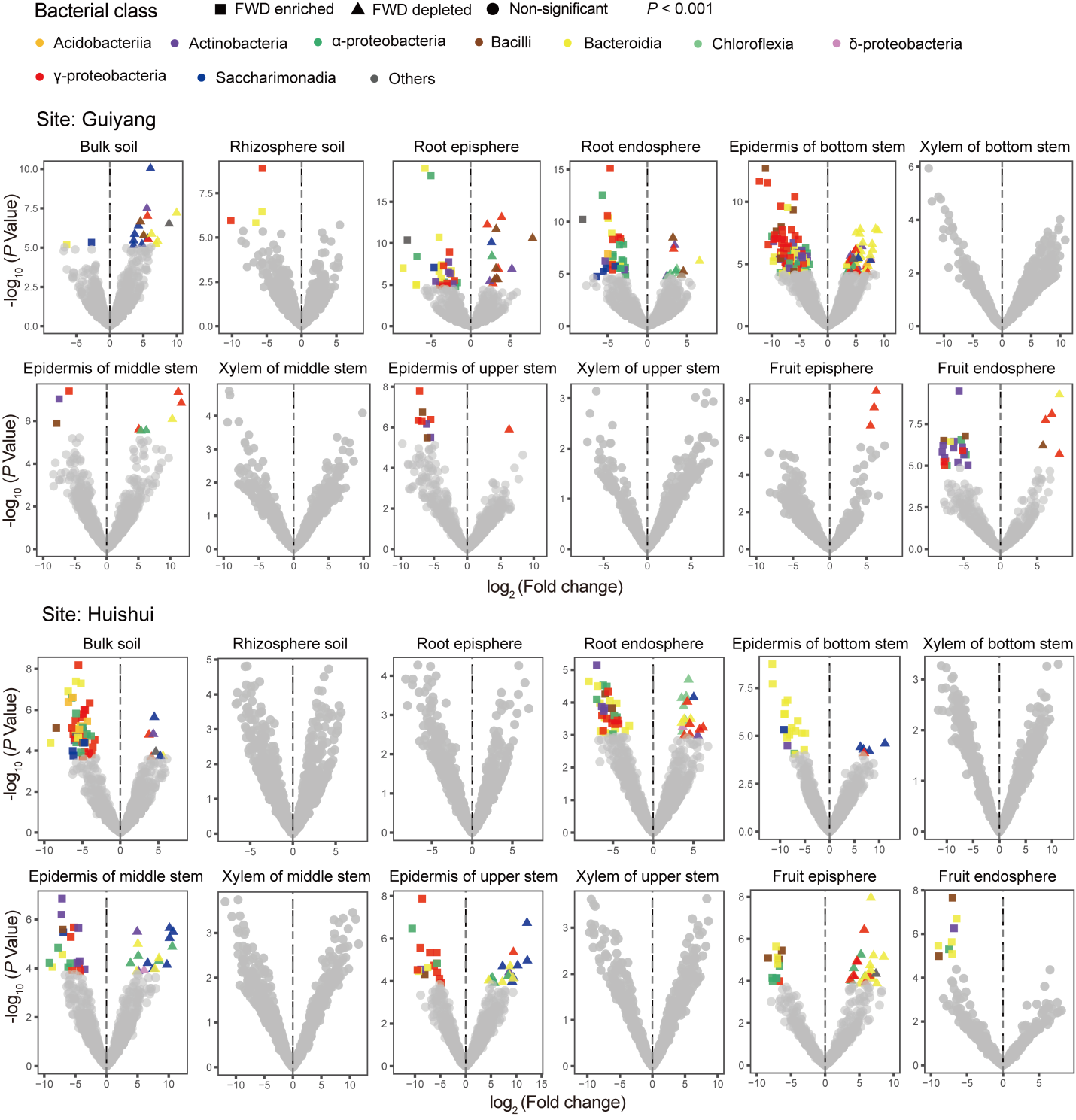
Fig. S8** The volcano plots illustrating the enrichment and depletion patterns of the bacterial class in diseased plants compartments at Guiyang (top) and Huishui (bottom), when the healthy plants were used as a baseline. The symbols correspond to FWD-enriched (square) and -depleted (triangle) ZOTUs.

**
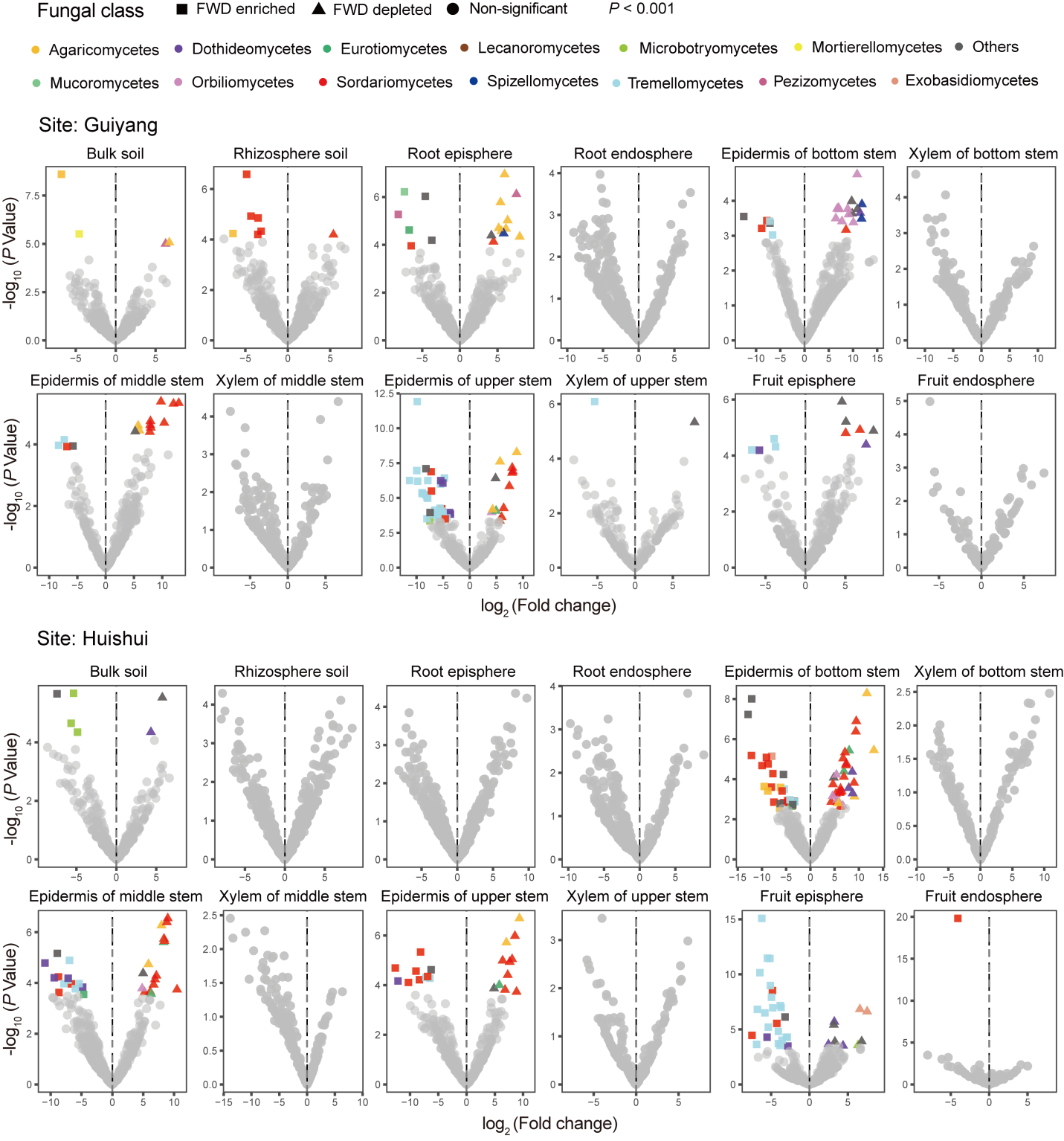
Fig. S9** The volcano plots illustrating the enrichment and depletion patterns of the fungal phylum in FWD plants compartments at Guiyang (top) and Huishui (bottom), when the healthy plants were used as a baseline. The symbols correspond to FWD-enriched (square) and -depleted (triangle) ZOTUs.


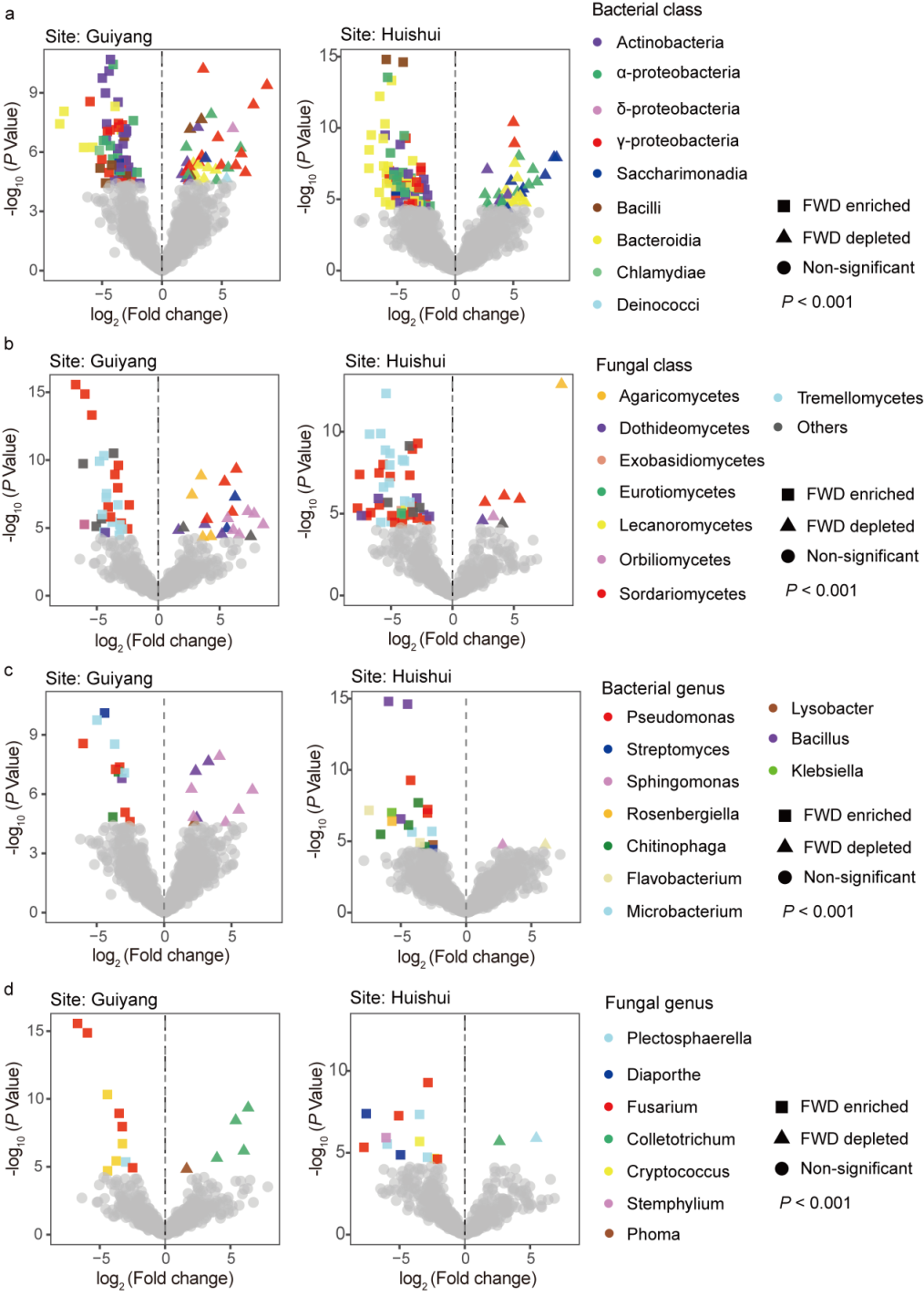
**Fig. S10** The volcano plots illustrating the enrichment and depletion patterns of microbiome in FWD plants all compartments at Guiyang (left) and Huishui (right), when the healthy plants were used as a baseline. The symbols correspond to FWD-enriched (square) and -depleted (triangle) ZOTUs. a–b Volcano plots showing FWD plants enriched and depleted bacterial and fungal class at Guiyang and Huishui, when the healthy plants were used as a baseline. c–d Volcano plots showing FWD plants enriched and depleted potentially beneficial bacteria and potentially pathogenic fungi at Guiyang and Huishui, when the healthy plants were used as a baseline.

**Fig. S11** Intra- and interkingdom co-occurrence networks at Guiyang and Huishui. **a** Networks analyses performed with the intra-kingdom of bacteria and fungi, and the interkingdom of bacteria and fungi together in healthy and FWD plant at Guiyang and Huishui. **b** Comparison of node-level topological features (degree and closeness centrality) in the corresponding interkingdom networks. The nodes were coloured according to the different kingdom (bacterial in blue and fungal in orange). **c** Numbers of bacterial–bacterial (BB), bacterial–fungal (BF), and fungal–fungal (FF) correlations in healthy and diseased plant interkingdom networks at Guiyang and Huishui. Green and red colors of the edges and column indicate positive and negative correlations, respectively. **d** Degree values of the healthy and diseased intra-kingdom networks at Guiyang and Huishui. The significance of difference was determined by nonparametric Kruskal–Wallis test. **e** Degree values of bacterial and fungal taxa in healthy and diseased interkingdom networks at Guiyang and Huishui. The significance of difference was determined by nonparametric Kruskal–Wallis test.


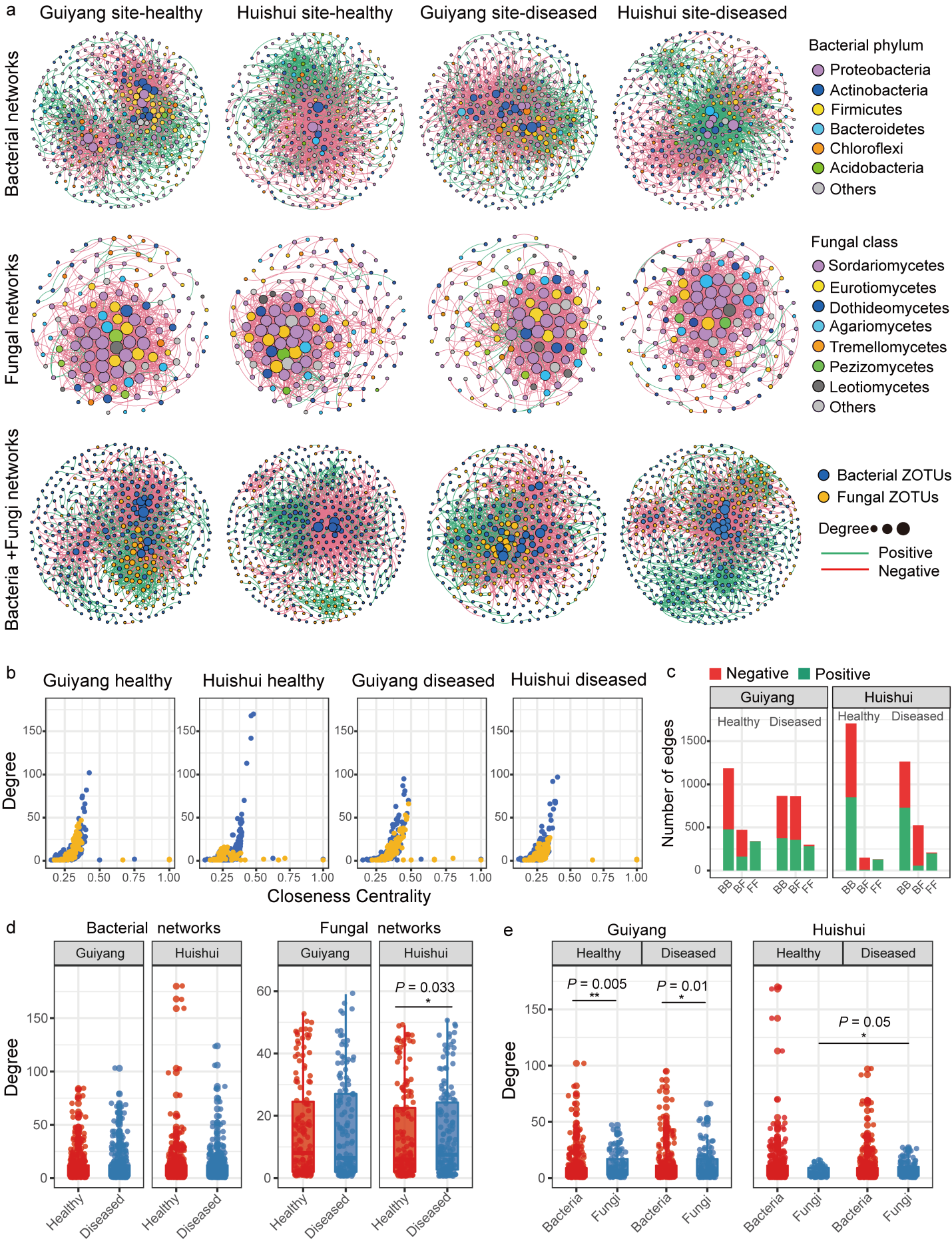


**Fig. S12** Interkingdom co-occurrence networks in soil, root, stem (3 sections), and fruit. **a** Networks performed with the kingdom of bacteria and fungi together in healthy (top) and diseased (bottom) soil, root, bottom stem, middle stem, upper stem, and fruit. The nodes were coloured according to the different kingdom (bacterial in blue, and fungi in orange). The sizes of the nodes are according to the degree of connection. The edges color represents positive (green) and negative (red) correlations, respectively. **b** Comparison of node-level topological features (degree and closeness centrality) of healthy (left) and diseased (right) network in the corresponding organ networks.


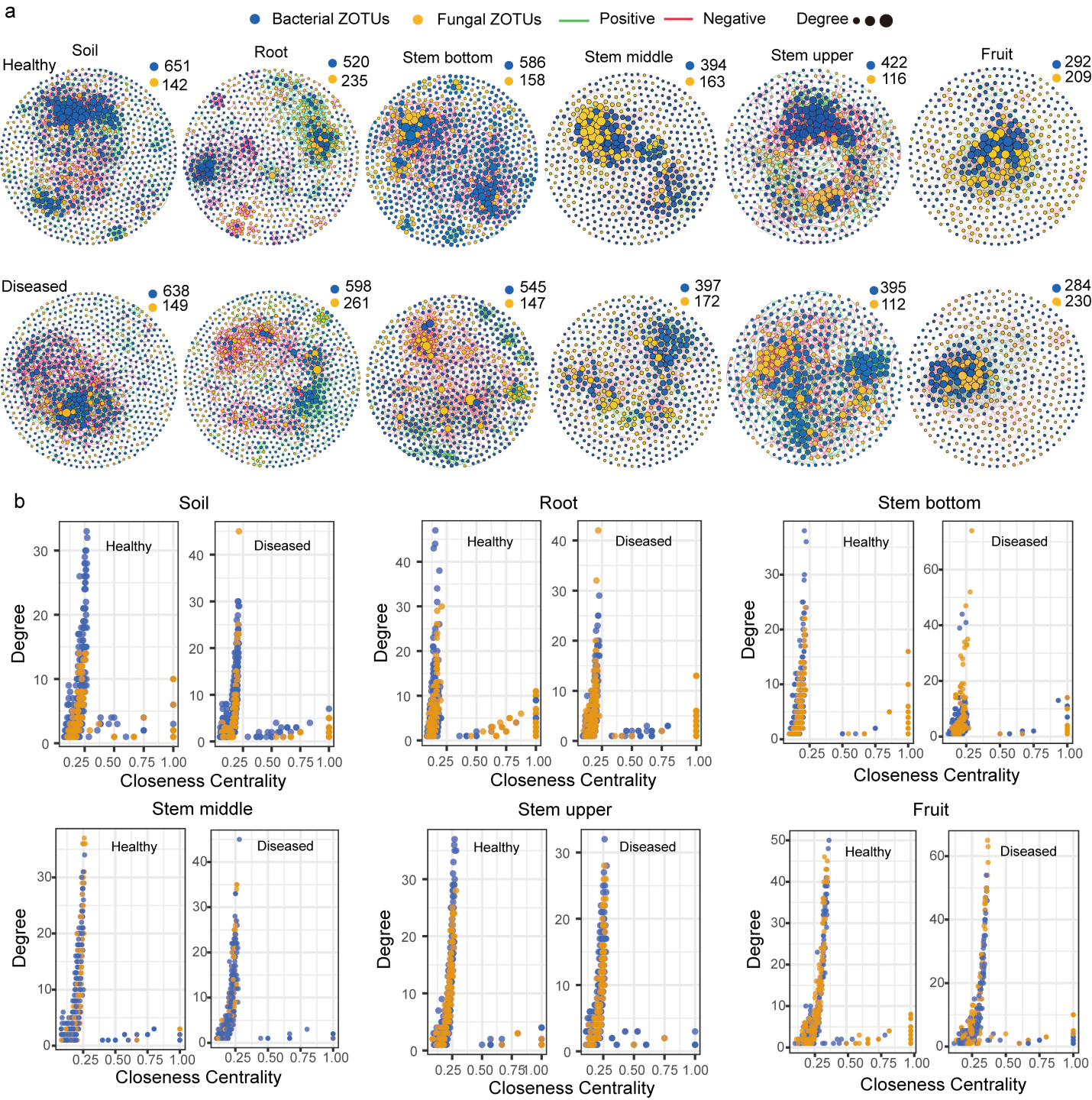


**
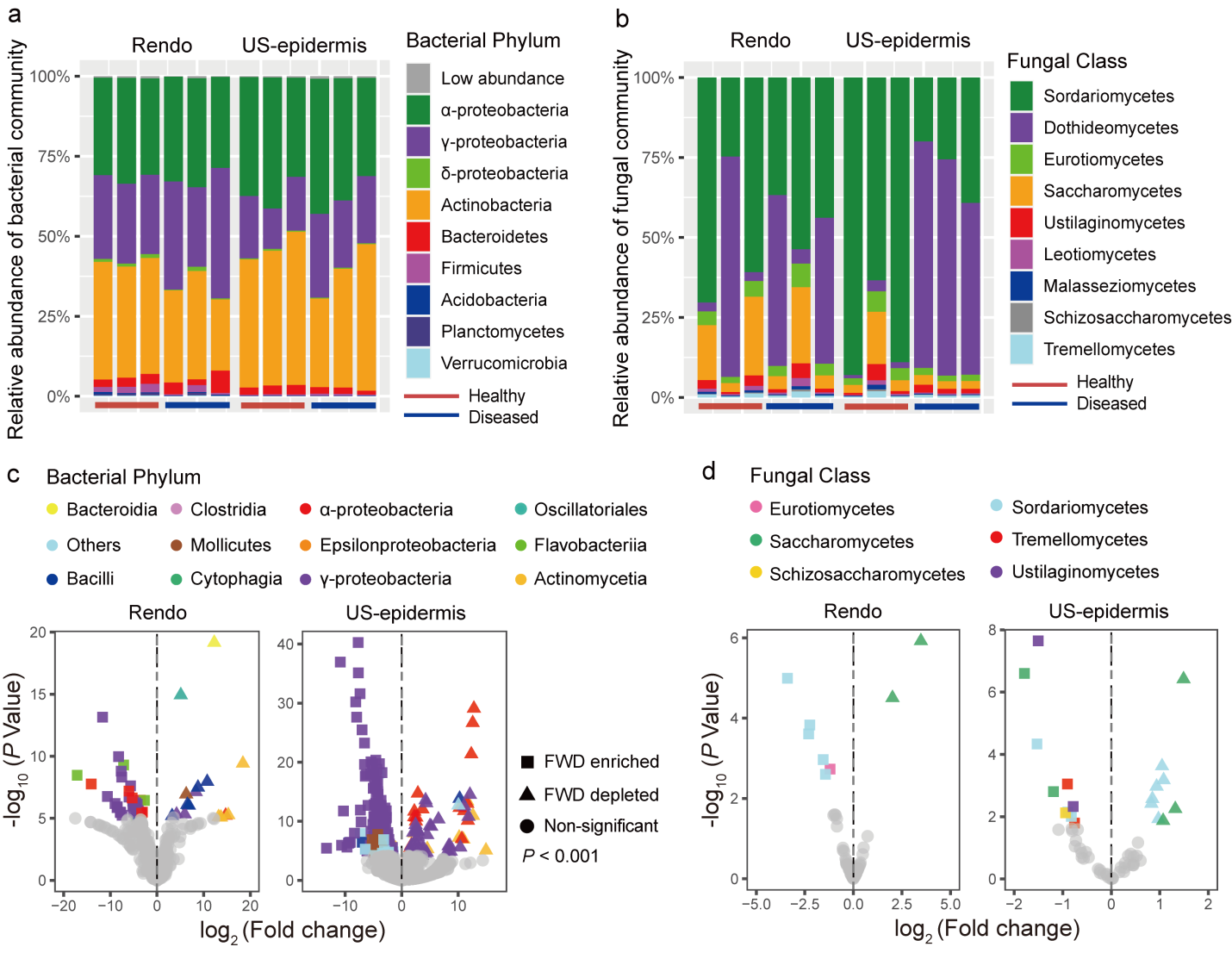
Fig. S13** Taxonomic composition and differentially abundant taxa of bacterial and fungal communities between the healthy and diseased root endosphere and upper stem epidermis from metagenomic sequencing data. Relative abundance of bacterial phyla (**a**) and fungal classes (**b**) varied between the healthy and diseased plant root endosphere and upper stem epidermis. **c** The volcano plots illustrating the enrichment and depletion patterns of bacterial phyla in diseased plant compared with the healthy plant. **d** The volcano plot illustrating the enrichment and depletion patterns of fungal classes in diseased plant compared with the healthy plant.


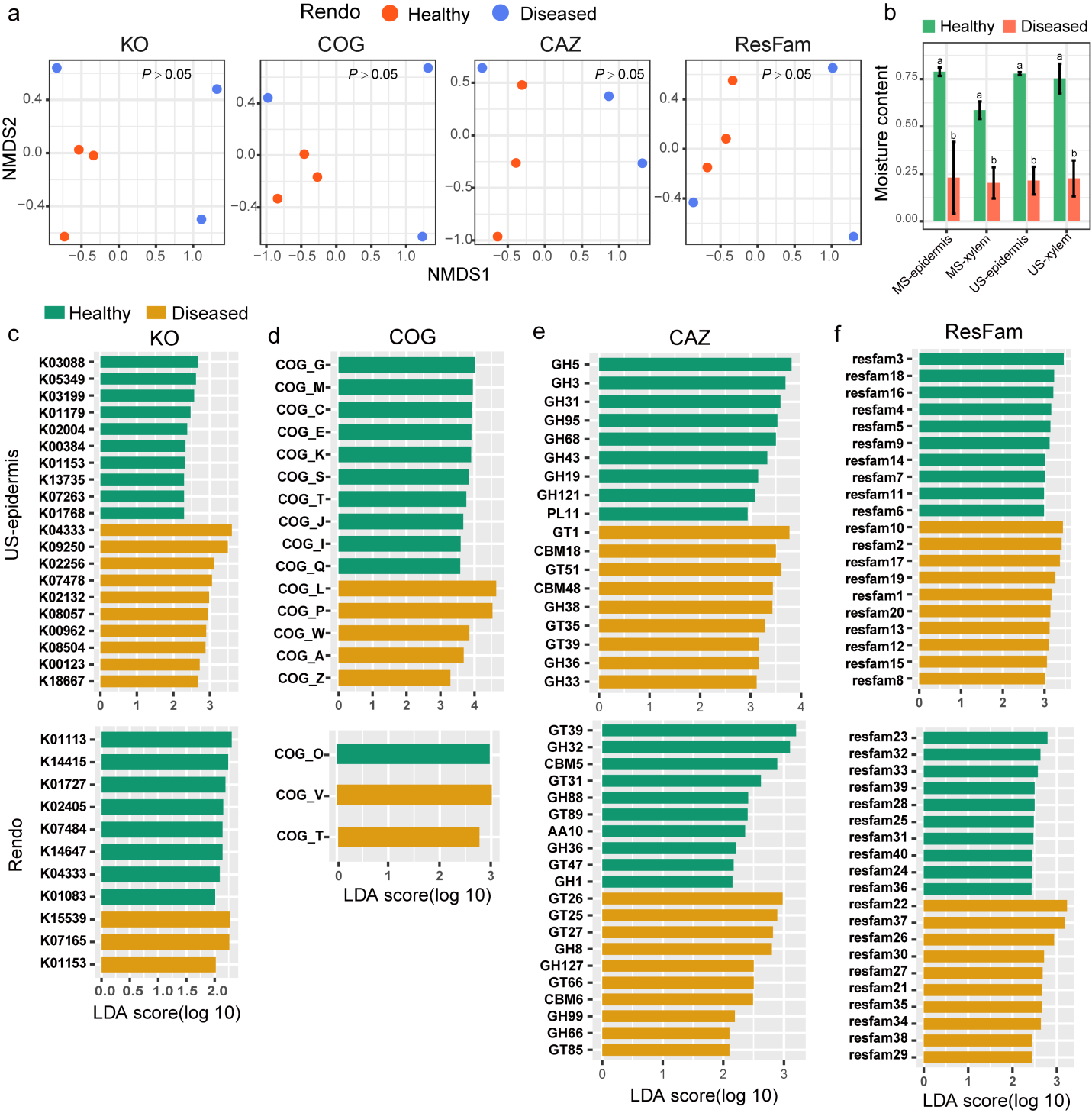
**Fig. S14** Changes of microbiome functional profiles between healthy and diseased root endosphere and upper stem epidermis. **a** NMDS ordinations based on Bray-Cutis distances matrices of healthy and diseased root endosphere compartment. **b** Moisture content in healthy and diseased middle and upper stem compartments. LEfSe difference analysis calculated the different functions of KO (**c**), COG (**d**), CAZ (**e**), and ResFam (**f**) profiles in US-epidermis (top) and Rendo (bottom).

**Supplementary tables**

**Table S1** Primers information used in this study.

| Gene fragment | Primers | Region | Sequence (5’-3’) | PCR program |
| --- | --- | --- | --- | --- |
| 16S rRNA | 799F | V5-V6 | AACMGGATTAGATACCCKG | 2 min initial denaturation at 94 °C, 30 cycles of 30 s at 94 °C, 30 s at 55 °C, and 45 s at 72 °C, with a final 10 min elongation at 72 °C. |
|  | 1115R |  | AGGGTTGCGCTCGTTG’ |  |
| ITS | fITS7 | ITS2 | GTGARTCATCGAATCTTTG | 5 min initial denaturation at 94 °C, 35 cycles of 30 s at 94 °C, 30 s at 56.5 °C, and 30 s at 72 °C, with a final 7 min elongation at 72 °C. |
|  | ITS4 |  | TCCTCCGCTTATTGATATGC |  |
| TEF | EF1 | - | ATGGGTAAGGARGACAAGAC | 1.5 min initial denaturation at 94 °C, 35 cycles of 45 s at 94 °C, 45 s at 55 °C, and 15 s at 72 °C, with a final 10 min elongation at 72 °C. |
|  | EF2 | - | GGARGTACCAGTSATCATG |  |
| RPB2 | RPB2-5f2 | - | GGGGWGAYCAGAAGAAGGC | 1.5 min initial denaturation at 94 °C, 35 cycles of 45 s at 94 °C, 45 s at 57 °C, and 20 s at 72 °C, with a final 10 min elongation at 72 °C. |
|  | RPB2-11ar | - | GCRTGGATCTTRTCRTCSACC |  |
| ITS | ITS1 | - | TCCGTAGGTGAACCTGCGG | 5 min initial denaturation at 95 °C, 35 cycles of 30 s at 95 °C, 30 s at 52 °C, and 10 s at 72 °C, with a final 10 min elongation at 72 °C. |
|  | ITS4 | - | TCCTCCGCTTATTGATATGC |  |

**Table S2** PERMANOVA by adonis of all bacterial 16S and fungal ITS samples. PERMANOVA analysis using the Bray Curtis distances for plant compartment, FWD, and sampling site in beta diversity.

| Microbial communities | Variables | Df^a^ | SumsOfSqs^b^ | MeanSqs^c^ | F.Model | *R*^2^ | Pr(>F)^d^ |
| --- | --- | --- | --- | --- | --- | --- | --- |
| Bacterial community | Compartment | 11 | 16.30 | 1.48 | 16.69 | 0.47 | 0.001 |
|  | FWD | 1 | 1.99 | 1.99 | 22.40 | 0.06 | 0.001 |
|  | Sampling site | 1 | 0.52 | 0.52 | 5.82 | 0.01 | 0.001 |
| Fungal community | Compartment | 11 | 17.60 | 1.60 | 19.53 | 0.53 | 0.001 |
|  | FWD | 1 | 1.07 | 1.07 | 13.06 | 0.03 | 0.001 |
|  | Sampling site | 1 | 0.69 | 0.69 | 8.36 | 0.02 | 0.001 |

^a^ degrees of freedom, ^b^ sum of squares, ^c^ mean sum of squares, ^d^ p-values are based on 999 permutations with subsequent Bonferroni correction.

**Table S3** PERMANOVA by adonis of bacterial 16S conducted separately for each compartment. PERMANOVA analysis using the BrayCurtis distances for sampling site and FWD performed separately at individual compartment.

| Compartment | Factor | Df^a^ | SumsOfSqs^b^ | MeanSqs^c^ | F.Model | *R*^2^ | Pr(>F)^d^ |
| --- | --- | --- | --- | --- | --- | --- | --- |
| BS | sampling site | 1 | 0.76801 | 0.76801 | 15.4205 | 0.54441 | 0.001 |
|  | FWD | 1 | 0.10404 | 0.10404 | 2.0889 | 0.07375 | 0.104 |
| RHS | sampling site | 1 | 0.75825 | 0.75825 | 15.4813 | 0.567 | 0.001 |
|  | FWD | 1 | 0.09556 | 0.09556 | 1.951 | 0.07146 | 0.095 |
| Repi | sampling site | 1 | 0.59354 | 0.59354 | 7.9493 | 0.41008 | 0.001 |
|  | FWD | 1 | 0.14462 | 0.14462 | 1.937 | 0.09992 | 0.099 |
| Rendo | sampling site | 1 | 0.47698 | 0.47698 | 7.26 | 0.37297 | 0.001 |
|  | FWD | 1 | 0.16537 | 0.16537 | 2.5171 | 0.12931 | 0.044 |
| BS-epidermis | sampling site | 1 | 0.50218 | 0.50218 | 7.1814 | 0.33163 | 0.001 |
|  | FWD | 1 | 0.29558 | 0.29558 | 4.2269 | 0.19519 | 0.004 |
| BS-xylem | sampling site | 1 | 0.43957 | 0.43957 | 2.7167 | 0.18556 | 0.001 |
|  | FWD | 1 | 0.39359 | 0.39359 | 2.4325 | 0.16615 | 0.002 |
| MS-epidermis | sampling site | 1 | 0.48414 | 0.48414 | 8.1686 | 0.37526 | 0.001 |
|  | FWD | 1 | 0.24243 | 0.24243 | 4.0904 | 0.18791 | 0.006 |
| MS-xylem | sampling site | 1 | 0.39485 | 0.39485 | 3.2207 | 0.21907 | 0.001 |
|  | FWD | 1 | 0.15622 | 0.15622 | 1.2742 | 0.08667 | 0.249 |
| US-epidermis | sampling site | 1 | 0.41999 | 0.41999 | 5.203 | 0.28178 | 0.001 |
|  | FWD | 1 | 0.30029 | 0.30029 | 3.7201 | 0.20147 | 0.002 |
| US-xylem | sampling site | 1 | 0.30718 | 0.30717 | 2.3315 | 0.18231 | 0.001 |
|  | FWD | 1 | 0.16582 | 0.16582 | 1.2586 | 0.09842 | 0.109 |
| Fepi | sampling site | 1 | 0.24192 | 0.241923 | 2.473 | 0.17108 | 0.001 |
|  | FWD | 1 | 0.19459 | 0.194586 | 1.9891 | 0.13761 | 0.014 |
| Fendo | sampling site | 1 | 0.20069 | 0.20069 | 1.952 | 0.14991 | 0.001 |
|  | FWD | 1 | 0.16602 | 0.16602 | 1.6148 | 0.12401 | 0.006 |

^a^ degrees of freedom, ^b^ sum of squares, ^c^ mean sum of squares, ^d^ p-values are based on 999 permutations with subsequent Bonferroni correction.

**Table S4** PERMANOVA by adonis of fungal ITS conducted separately for each compartment. PERMANOVA analysis using the Bray Curtis distances for sampling site and FWD performed separately at individual compartment.

| Compartment | Factor | Df^a^ | SumsOfSqs^b^ | MeanSqs^c^ | F.Model | *R*^2^ | Pr(>F)^d^ |
| --- | --- | --- | --- | --- | --- | --- | --- |
| BS | sampling site | 1 | 0.36364 | 0.36364 | 7.0055 | 0.39483 | 0.001 |
|  | FWD | 1 | 0.06774 | 0.06774 | 1.3051 | 0.07355 | 0.121 |
| RHS | sampling site | 1 | 0.38697 | 0.38697 | 7.7881 | 0.41769 | 0.001 |
|  | FWD | 1 | 0.0724 | 0.0724 | 1.4571 | 0.07815 | 0.086 |
| Repi | sampling site | 1 | 0.54273 | 0.54273 | 7.3841 | 0.3995 | 0.001 |
|  | FWD | 1 | 0.12678 | 0.12678 | 1.7249 | 0.09332 | 0.090 |
| Rendo | sampling site | 1 | 0.2256 | 0.2256 | 2.5612 | 0.16107 | 0.025 |
|  | FWD | 1 | 0.3456 | 0.3456 | 3.9236 | 0.24675 | 0.001 |
| BS-epidermis | sampling site | 1 | 0.38531 | 0.38531 | 4.0346 | 0.22101 | 0.001 |
|  | FWD | 1 | 0.42602 | 0.42602 | 4.461 | 0.24437 | 0.001 |
| BS-xylem | sampling site | 1 | 0.22986 | 0.22986 | 1.9657 | 0.14733 | 0.016 |
|  | FWD | 1 | 0.21043 | 0.21043 | 1.7995 | 0.13488 | 0.034 |
| MS-epidermis | sampling site | 1 | 0.37868 | 0.37868 | 4.6261 | 0.24715 | 0.001 |
|  | FWD | 1 | 0.35324 | 0.35324 | 4.3153 | 0.23055 | 0.001 |
| MS-xylem | sampling site | 1 | 0.26597 | 0.26597 | 2.2605 | 0.15601 | 0.001 |
|  | FWD | 1 | 0.24822 | 0.24822 | 2.1096 | 0.14559 | 0.003 |
| US-epidermis | sampling site | 1 | 0.1934 | 0.1934 | 2.5331 | 0.1325 | 0.055 |
|  | FWD | 1 | 0.56964 | 0.56964 | 7.4611 | 0.39026 | 0.001 |
| US-xylem | sampling site | 1 | 0.20039 | 0.200392 | 2.0901 | 0.1536 | 0.001 |
|  | FWD | 1 | 0.20787 | 0.207871 | 2.1681 | 0.15933 | 0.001 |
| Fepi | sampling site | 1 | 0.23726 | 0.237262 | 3.9495 | 0.2446 | 0.001 |
|  | FWD | 1 | 0.14984 | 0.14984 | 2.4943 | 0.15447 | 0.001 |
| Fendo | sampling site | 1 | 0.1046 | 0.104598 | 1.3772 | 0.11941 | 0.004 |
|  | FWD | 1 | 0.08504 | 0.085043 | 1.1197 | 0.09709 | 0.132 |

^a^ degrees of freedom, ^b^ sum of squares, ^c^ mean sum of squares, ^d^ p-values are based on 999 permutations with subsequent Bonferroni correction.

**Table S5** Distance to centroid was calculated by analysis of beta dispersion using Bray-Curtis dissimilarity.

| Community dissimilarity | | Bacterial | | Fungal | |
| --- | --- | --- | --- | --- | --- |
| Compartments | Condition | Average distance to centroid | Pr (>F) | Average distance to centroid | Pr (>F) |
| BS | Healthy | 0.189 | 0.014* | 0.2297 | 0.366 |
|  | Diseased | 0.220 |  | 0.2403 |  |
| RHS | Healthy | 0.1687 | 0.539 | 0.1999 | 0.016* |
|  | Diseased | 0.1724 |  | 0.2367 |  |
| Repi | Healthy | 0.1901 | 0.211 | 0.2367 | 0.067 |
|  | Diseased | 0.2137 |  | 0.2739 |  |
| Rendo | Healthy | 0.1860 | 0.013* | 0.2348 | 0.459 |
|  | Diseased | 0.2263 |  | 0.2549 |  |
| BS-epidermis | Healthy | 0.2061 | 0.025* | 0.3217 | 0.527 |
|  | Diseased | 0.2563 |  | 0.2914 |  |
| BS-xylem | Healthy | 0.2908 | 0.005** | 0.3299 | 0.767 |
|  | Diseased | 0.3597 |  | 0.3122 |  |
| MS-epidermis | Healthy | 0.2283 | 0.226 | 0.2599 | 0.123 |
|  | Diseased | 0.2444 |  | 0.2899 |  |
| MS-xylem | Healthy | 0.2679 | 0.41 | 0.2104 | 0.001*** |
|  | Diseased | 0.2924 |  | 0.3112 |  |
| US-epidermis | Healthy | 0.2266 | 0.007** | 0.1741 | 0.001*** |
|  | Diseased | 0.2445 |  | 0.2823 |  |
| US-xylem | Healthy | 0.2500 | 0.078 | 0.2098 | 0.151 |
|  | Diseased | 0.3167 |  | 0.2547 |  |
| Fepi | Healthy | 0.2170 | 0.001*** | 0.2383 | 0.016* |
|  | Diseased | 0.2797 |  | 0.2068 |  |
| Fendo | Healthy | 0.2460 | 0.264 | 0.1617 | 0.936 |
|  | Diseased | 0.2184 |  | 0.1628 |  |

**Table S6** Linear-mixed models (LMMs) for alpha diversity indices. Effects of plant compartment, sampling site and FWD on bacterial and fungal community alpha diversity indices were tested with linear-mixed models (LMMs). Significance was assessed using type II ANOVA with Kenward–Rodger approximation of the degrees of freedom in a linear-mixed model.

| Microbial communities | Variables | Shannon diversity | | Chao1 richness | |
| --- | --- | --- | --- | --- | --- |
|  |  | *F* value | *P* ( > *F*) | *F* value | *P* ( > *F*) |
| Bacterial community | Compartment | F11,121= 14.9 | < 2.2e-16 | F11,121= 71.93 | < 2.2e-16 |
|  | FWD | F1,9 = 7.4 | 0.023 | F1,9 = 9.69 | < 0.012 |
|  | Sampling site | F1,9 = 12.1 | 0.006 | F1,9 = 17.49 | 0.002 |
| Fungal community | Compartment | F11,121 = 7.9 | < 3.24e-10 | F11,121 = 24.39 | < 2.2e-16 |
|  | FWD | F1, 9 = 19.4 | 0.00172 | F1, 9 = 17.97 | 0.002 |
|  | Sampling site | F1, 9 = 0.048 | 0.831 | F1, 9 = 5.46 | 0.044 |

**Table S7** Linear-mixed model for bacterial phylum and fungal class composition. Effects of plant compartment, sampling site and FWD on bacterial phylum and fungal class composition were tested with LMM. Significance was assessed using type II ANOVA with Kenward–Rodger approximation of the degrees of freedom in a linear-mixed model.

| Microbial communities | Variables | Plant compartment | | FWD | | Sampling site | |
| --- | --- | --- | --- | --- | --- | --- | --- |
|  | Microbial composition | *F* value | *P* ( > *F*) | *F* value | *P* ( > *F*) | *F* value | *P* ( > *F*) |
| Bacterial community | Proteobacteria.Gammaproteobacteria | 1.89 | 0.047 | 0.005 | 0.94 | 0.01 | 0.91 |
|  | Proteobacteria.Alphaproteobacteria | 5.30 | 8.338e-07 | 0.045 | 0.94 | 1.32 | 0.27 |
|  | Proteobacteria.Deltaproteobacteria | 22.71 | <2e-16 | 3.37 | 0.09 | 1.25 | 0.29 |
|  | Actinobacteria | 4.38 | 1.589e-05 | 2.42 | 0.15 | 2.04 | 0.19 |
|  | Bacteroidetes | 2.13 | 0.02 | 0.12 | 0.91 | 1.51 | 0.25 |
|  | Firmicutes | 9.69 | 2.191e-12 | 0.89 | 0.37 | 4.05 | 0.07 |
|  | Patescibacteria | 11.14 | 4.904e-14 | 0.12 | 0.91 | 21.90 | 0.001 |
|  | Chloroflexi | 15.81 | < 2.2e-16 | 2.37 | 0.15 | 19.14 | 0.002 |
|  | Gemmatimonadetes | 64.79 | <2e-16 | 0.029 | 0.60 | 0.49 | 0.5 |
|  | Acidobacteria | 28.25 | <2e-16 | 1.21 | 0.30 | 1.93 | 0.19 |
| Fungal community | Ascomycota.Sordariomycetes | 1.45 | 0.15 | 0.59 | 0.46 | 3.8 | 0.08 |
|  | Ascomycota.Dothideomycetes | 14.19 | <2e-16 | 2.22 | 0.17 | 0.15 | 0.69 |
|  | Fungi.unidentified | 5.1 | 1.575e-06 | 0.10 | 0.75 | 8.33 | 0.01 |
|  | Basidiomycota.Tremellomycetes | 6.42 | 2.548e-08 | 8.94 | 0.01 | 1.41 | 0.27 |
|  | Ascomycota.unidentified | 4.39 | 1.545e-05 | 6.66 | 0.029 | 0.25 | 0.63 |
|  | Ascomycota.Eurotiomycetes | 12.64 | 1.238e-15 | 4.37 | 0.06 | 1.91 | 0.20 |
|  | Ascomycota.Pezizomycetes | 1.46 | 0.15 | 0.21 | 0.65 | 2.87 | 0.12 |
|  | Basidiomycota.Agaricomycetes | 0.92 | 0.52 | 0.42 | 0.53 | 1.69 | 0.23 |
|  | Ascomycota.Saccharomycetes | 1.03 | 0.42 | 1.01 | 0.34 | 0.97 | 0.35 |
|  | Low abundance | 4.65 | 6.738e-06 | 1.07 | 0.32 | 2.03 | 0.18 |
|  | Ascomycota.Orbiliomycetes | 3.66 | 0.00016 | 4.04 | 0.07 | 4.10 | 0.07 |
|  | Mortierellomycota.Mortierellomycetes | 14.73 | < 2.2e-16 | 0.18 | 0.68 | 14.63 | 0.0041 |
|  | Ascomycota.Leotiomycetes | 1.45 | 0.16 | 0.79 | 0.39 | 1.7177 | 0.22 |

**Table S8** Differentially abundant analysis showing the enrichment and depletion patterns of bacterial taxa in diseased organs compared with healthy organs.

| Plant organ | Treatment | ZOTUs ID | Taxonomic position | *P* value |
| --- | --- | --- | --- | --- |
| Soil | Enriched (n=2) | ZOTU531 | Bacteria--Bacteroidetes--Bacteroidia--Chitinophagales--Chitinophagaceae--uncultured | 6.75E-07 |
|  |  | ZOTU999 | Bacteria--Proteobacteria--Alphaproteobacteria--Micropepsales--Micropepsaceae--uncultured | 6.28E-07 |
|  | Depleted (n=0) | -- | -- | -- |
| Root | Enriched (n=33) | ZOTU55 | Bacteria--Bacteroidetes--Bacteroidia--Flavobacteriales--Weeksellaceae--Chryseobacterium | 2.77E-10 |
|  |  | ZOTU431 | Bacteria--Bacteroidetes--Bacteroidia--Flavobacteriales--Weeksellaceae--Chryseobacterium | 5.63E-09 |
|  |  | ZOTU223 | Bacteria--Bacteroidetes--Bacteroidia--Chitinophagales--Chitinophagaceae--Chitinophaga | 6.59E-08 |
|  |  | ZOTU274 | Bacteria--Proteobacteria--Alphaproteobacteria--Rhizobiales--Devosiaceae--Devosia | 1.48E-07 |
|  |  | ZOTU278 | Bacteria--Proteobacteria--Alphaproteobacteria--Rhizobiales--Devosiaceae--Devosia | 1.88E-07 |
|  |  | ZOTU1470 | Bacteria--Bacteroidetes--Bacteroidia--Chitinophagales--Chitinophagaceae--Chitinophaga | 2.75E-07 |
|  |  | ZOTU226 | Bacteria--Bacteroidetes--Bacteroidia--Chitinophagales--Chitinophagaceae--Taibaiella | 2.83E-07 |
|  |  | ZOTU947 | Bacteria--Bacteroidetes--Bacteroidia--Sphingobacteriales--Sphingobacteriaceae--Pedobacter | 3.13E-07 |
|  |  | ZOTU154 | Bacteria--Bacteroidetes--Bacteroidia--Chitinophagales--Chitinophagaceae--Taibaiella | 3.34E-07 |
|  |  | ZOTU3425 | Bacteria--Bacteroidetes--Bacteroidia--Chitinophagales--Chitinophagaceae--Taibaiella | 6.35E-07 |
|  | Depleted (n=3) | ZOTU1036 | Bacteria--Chloroflexi--Chloroflexia--Chloroflexales--Roseiflexaceae--uncultured | 1.23E-06 |
|  |  | ZOTU122 | Bacteria--Proteobacteria--Gammaproteobacteria--Betaproteobacteriales--Burkholderiaceae--Comamonas | 9.73E-06 |
|  |  | ZOTU2742 | Bacteria--Actinobacteria--Actinobacteria--Frankiales--uncultured--unculturedbacterium | 9.90E-06 |
| Stem | Enriched(n=152) | ZOTU52 | Bacteria--Proteobacteria--Gammaproteobacteria--Pseudomonadales--Pseudomonadaceae--Pseudomonas | 7.03E-21 |
|  |  | ZOTU58 | Bacteria--Proteobacteria--Gammaproteobacteria--Enterobacteriales--Enterobacteriaceae--Pantoea | 7.10E-17 |
|  |  | ZOTU30 | Bacteria--Firmicutes--Bacilli--Bacillales--Bacillaceae--Bacillus | 7.01E-16 |
|  |  | ZOTU92 | Bacteria--Proteobacteria--Gammaproteobacteria--Enterobacteriales--Enterobacteriaceae--Pantoea | 9.02E-16 |
|  |  | ZOTU2 | Bacteria--Actinobacteria--Actinobacteria--Streptomycetales--Streptomycetaceae--Streptomyces | 3.66E-15 |
|  |  | ZOTU19 | Bacteria--Actinobacteria--Actinobacteria--Micrococcales--Microbacteriaceae--Microbacterium | 2.36E-14 |
|  |  | ZOTU116 | Bacteria--Firmicutes--Bacilli--Bacillales--Bacillaceae--Bacillus | 3.18E-14 |
|  |  | ZOTU247 | Bacteria--Bacteroidetes--Bacteroidia--Sphingobacteriales--Sphingobacteriaceae--Sphingobacterium | 4.34E-13 |
|  |  | ZOTU139 | Bacteria--Proteobacteria--Alphaproteobacteria--Rhodobacterales--Rhodobacteraceae--Falsirhodobacter | 7.12E-13 |
|  |  | ZOTU59 | Bacteria--Bacteroidetes--Bacteroidia--Sphingobacteriales--Sphingobacteriaceae--Sphingobacterium | 2.05E-12 |
|  | Depleted (n=203) | ZOTU602 | Bacteria--Proteobacteria--Gammaproteobacteria--Betaproteobacteriales--Burkholderiaceae--Massilia | 1.04E-13 |
|  |  | ZOTU270 | Bacteria--Patescibacteria--Saccharimonadia--Saccharimonadales--Saccharimonadaceae--unculturedbacterium | 3.25E-13 |
|  |  | ZOTU800 | Bacteria--Patescibacteria--Saccharimonadia--Saccharimonadales--Saccharimonadaceae--unculturedbacterium | 1.14E-12 |
|  |  | ZOTU466 | Bacteria--Patescibacteria--Saccharimonadia--Saccharimonadales--Saccharimonadaceae--unculturedbacterium | 2.26E-11 |
|  |  | ZOTU462 | Bacteria--Bacteroidetes--Bacteroidia--Chitinophagales--Chitinophagaceae--Parasegetibacter | 3.60E-11 |
|  |  | ZOTU172 | Bacteria--Proteobacteria--Alphaproteobacteria--Sphingomonadales--Sphingomonadaceae--Sphingomonas | 4.02E-11 |
|  |  | ZOTU94 | Bacteria--Patescibacteria--Saccharimonadia--Saccharimonadales--candidatedivisionTM7bacteriumJGI0001002-L20--candidatedivisionTM7bacteriumJGI0001002-L20 | 1.23E-10 |
|  |  | ZOTU28 | Bacteria--Proteobacteria--Gammaproteobacteria--Betaproteobacteriales--Burkholderiaceae--Acidovorax | 1.47E-10 |
|  |  | ZOTU322 | Bacteria--Proteobacteria--Alphaproteobacteria--Rhizobiales--Beijerinckiaceae--Methylobacterium | 1.93E-10 |
|  |  | ZOTU485 | Bacteria--Bacteroidetes--Bacteroidia--Cytophagales--Hymenobacteraceae--Hymenobacter | 2.19E-10 |
| Fruit | Enriched(n=24) | ZOTU16 | Bacteria--Proteobacteria--Gammaproteobacteria--Pseudomonadales--Pseudomonadaceae--Pseudomonas | 1.23E-09 |
|  |  | ZOTU82 | Bacteria--Proteobacteria--Alphaproteobacteria--Sphingomonadales--Sphingomonadaceae--Sphingomonas | 1.66E-08 |
|  |  | ZOTU95 | Bacteria--Actinobacteria--Actinobacteria--Micrococcales--Microbacteriaceae--Leucobacter | 2.63E-08 |
|  |  | ZOTU394 | Bacteria--Actinobacteria--Actinobacteria--Micrococcales--Microbacteriaceae--Agrococcus | 2.65E-07 |
|  |  | ZOTU119 | Bacteria--Actinobacteria--Actinobacteria--Micrococcales--Beutenbergiaceae--Salana | 3.34E-07 |
|  |  | ZOTU264 | Bacteria--Actinobacteria--Actinobacteria--Micrococcales--Microbacteriaceae--Curtobacterium | 3.66E-07 |
|  |  | ZOTU13 | Bacteria--Proteobacteria--Gammaproteobacteria--Enterobacteriales--Enterobacteriaceae--Klebsiella | 3.72E-07 |
|  |  | ZOTU139 | Bacteria--Proteobacteria--Alphaproteobacteria--Rhodobacterales--Rhodobacteraceae--Falsirhodobacter | 4.12E-07 |
|  |  | ZOTU296 | Bacteria--Proteobacteria--Alphaproteobacteria--Rhizobiales--Beijerinckiaceae--Methylobacterium | 5.45E-07 |
|  |  | ZOTU59 | Bacteria--Bacteroidetes--Bacteroidia--Sphingobacteriales--Sphingobacteriaceae--Sphingobacterium | 6.24E-07 |
|  | Depleted (n=9) | ZOTU183 | Bacteria--Proteobacteria--Gammaproteobacteria--Betaproteobacteriales--Burkholderiaceae--Pusillimonas | 2.09E-09 |
|  |  | ZOTU353 | Bacteria--Proteobacteria--Gammaproteobacteria--Enterobacteriales--Enterobacteriaceae--Pantoea | 2.54E-09 |
|  |  | ZOTU56 | Bacteria--Bacteroidetes--Bacteroidia--Sphingobacteriales--Sphingobacteriaceae--Sphingobacterium | 8.19E-08 |
|  |  | ZOTU43 | Bacteria--Proteobacteria--Gammaproteobacteria--Enterobacteriales--Enterobacteriaceae--Pantoea | 5.67E-07 |
|  |  | ZOTU9 | Bacteria--Proteobacteria--Gammaproteobacteria--Xanthomonadales--Xanthomonadaceae--Stenotrophomonas | 9.27E-07 |
|  |  | ZOTU527 | Bacteria--Proteobacteria--Alphaproteobacteria--Rhizobiales--Rhizobiaceae--Allorhizobium-Neorhizobium-Pararhizobium-Rhizobium | 1.49E-06 |
|  |  | ZOTU680 | Bacteria--Proteobacteria--Gammaproteobacteria--Pseudomonadales--Moraxellaceae--Acinetobacter | 1.70E-06 |
|  |  | ZOTU5 | Bacteria--Proteobacteria--Alphaproteobacteria--Rhizobiales--Rhizobiaceae--Allorhizobium-Neorhizobium-Pararhizobium-Rhizobium | 5.11E-06 |
|  |  | ZOTU242 | Bacteria--Bacteroidetes--Bacteroidia--Flavobacteriales--Weeksellaceae--Chryseobacterium | 1.11E-05 |

**Table S9** Differentially abundant analysis showing the enrichment and depletion patterns of fungal taxa in diseased organs compared with healthy organs.

| Plant organ | Treatment | ZOTUs ID | Taxonomic position | *P* value |
| --- | --- | --- | --- | --- |
| Soil | Enriched (n=2) | ZOTU18 | Fungi--Ascomycota--Pezizomycetes--Pezizales--unidentified--unidentified | 9.01E-07 |
|  |  | ZOTU283 | Fungi--Glomeromycota--unidentified--unidentified--unidentified--unidentified | 5.80E-07 |
|  | Depleted (n=0) | -- | -- | -- |
| Root | Enriched (n=11) | ZOTU15 | Fungi--Ascomycota--Sordariomycetes--Hypocreales--Nectriaceae--Fusarium | 4.42E-08 |
|  |  | ZOTU8 | Fungi--Ascomycota--Eurotiomycetes--Chaetothyriales--Herpotrichiellaceae--Exophiala | 8.52E-08 |
|  |  | ZOTU22 | Fungi--Ascomycota--Sordariomycetes--Sordariales--Chaetomiaceae--Chaetomium | 1.63E-07 |
|  |  | ZOTU330 | Fungi--Ascomycota--Pezizomycetes--Pezizales--Sarcosomataceae--unidentified | 1.31E-06 |
|  |  | ZOTU6 | Fungi--Ascomycota--unidentified--unidentified--unidentified--unidentified | 1.58E-06 |
|  |  | ZOTU18 | Fungi--Ascomycota--Pezizomycetes--Pezizales--unidentified--unidentified | 2.13E-06 |
|  |  | ZOTU48 | Fungi--Ascomycota--Sordariomycetes--Glomerellales--Plectosphaerellaceae--Plectosphaerella | 2.57E-06 |
|  |  | ZOTU60 | Fungi--Ascomycota--Sordariomycetes--Hypocreales--Nectriaceae--Fusarium | 2.58E-06 |
|  |  | ZOTU203 | Fungi--Ascomycota--Sordariomycetes--Hypocreales--Bionectriaceae--Clonostachys | 4.45E-06 |
|  |  | ZOTU113 | Fungi--Ascomycota--Sordariomycetes--Hypocreales--Hypocreaceae--Trichoderma | 5.04E-06 |
|  | Depleted (n=0) | -- | -- | -- |
| Stem | Enriched(n=59) | ZOTU61 | Fungi--Basidiomycota--Tremellomycetes--Tremellales--Bulleribasidiaceae--Hannaella | 4.00E-14 |
|  |  | ZOTU58 | Fungi--Basidiomycota--Tremellomycetes--Tremellales--Bulleribasidiaceae--Hannaella | 1.79E-13 |
|  |  | ZOTU29 | Fungi--Basidiomycota--Tremellomycetes--Tremellales--Bulleribasidiaceae--Hannaella | 1.84E-13 |
|  |  | ZOTU42 | Fungi--Basidiomycota--Tremellomycetes--Tremellales--Bulleribasidiaceae--Hannaella | 4.10E-13 |
|  |  | ZOTU10 | Fungi--Ascomycota--Sordariomycetes--Hypocreales--Nectriaceae--Fusarium | 9.60E-13 |
|  |  | ZOTU80 | Fungi--Basidiomycota--Tremellomycetes--Tremellales--Bulleribasidiaceae--Hannaella | 3.43E-12 |
|  |  | ZOTU69 | Fungi--Basidiomycota--Tremellomycetes--Tremellales--Bulleribasidiaceae--Vishniacozyma | 2.62E-11 |
|  |  | ZOTU102 | Fungi--Ascomycota--Sordariomycetes--Diaporthales--Diaporthaceae--Diaporthe | 2.01E-09 |
|  |  | ZOTU26 | Fungi--Basidiomycota--Tremellomycetes--Filobasidiales--Filobasidiaceae--Filobasidium | 2.22E-09 |
|  |  | ZOTU37 | Fungi--unidentified--unidentified--unidentified--unidentified--unidentified | 2.51E-09 |
|  | Depleted (n=203) | ZOTU996 | Fungi--Ascomycota--Sordariomycetes--Diaporthales--unidentified--unidentified | 6.22E-18 |
|  |  | ZOTU21 | Fungi--Basidiomycota--Agaricomycetes--Cantharellales--Ceratobasidiaceae--Thanatephorus | 1.87E-16 |
|  |  | ZOTU563 | Fungi--Ascomycota--Sordariomycetes--Diaporthales--Valsaceae--unidentified | 9.27E-16 |
|  |  | ZOTU241 | Fungi--Ascomycota--Eurotiomycetes--Chaetothyriales--Herpotrichiellaceae--Minimelanolocus | 1.20E-15 |
|  |  | ZOTU856 | Fungi--Ascomycota--Sordariomycetes--Sordariales--Chaetomiaceae--Zopfiella | 1.65E-15 |
|  |  | ZOTU115 | Fungi--Ascomycota--Sordariomycetes--Sordariales--Lasiosphaeriaceae--Cercophora | 2.22E-14 |
|  |  | ZOTU300 | Fungi--Ascomycota--Sordariomycetes--Microascales--Microascaceae--Petriellopsis | 1.27E-13 |
|  |  | ZOTU767 | Fungi--Ascomycota--Sordariomycetes--Sordariales--Lasiosphaeriaceae--Podospora | 3.47E-13 |
|  |  | ZOTU51 | Fungi--Ascomycota--Sordariomycetes--Glomerellales--Glomerellaceae--Colletotrichum | 4.28E-13 |
|  |  | ZOTU1050 | Fungi--Ascomycota--Sordariomycetes--Sordariales--Lasiosphaeriaceae--Podospora | 7.39E-13 |
| Fruit | Enriched(n=0) | -- | -- | -- |
|  | Depleted (n=0) | -- | -- | -- |

**Table S10** Topology properties of the intra- and interkingdom networks.

| Networks | No. Node | No. positive edges/proportion (%) | No. negative edges/proportion (%) | Clustering coefficient | Avg. degree | Modularity | Network density |
| --- | --- | --- | --- | --- | --- | --- | --- |
| Intra-Bacterial-H (Guiyang) | 407 | 1000/50% | 1000/50% | 0.344 | 9.828 | 0.482 | 0.024 |
| Intra-Bacterial-H (Huishui) | 383 | 1000/50% | 999/50% | 0.404 | 10.439 | 0.452 | 0.027 |
| Intra-Bacterial-D (Guiyang) | 391 | 1000/50% | 1000/50% | 0.295 | 10.23 | 0.380 | 0.026 |
| Intra-Bacterial-D (Huishui) | 395 | 1000/51.2% | 953/48.8% | 0.318 | 9.889 | 0.440 | 0.025 |
| Intra-Bacterial-H | 336 | 1000/62.2% | 608/37.8% | 0.419 | 9.571 | 0.464 | 0.029 |
| Intra-Bacterial-D | 324 | 1000/80.1% | 249/19.9% | 0.325 | 7.71 | 0.501 | 0.024 |
| Intra-Fungal-H (Guiyang) | 139 | 1000/94.6% | 57/5.4% | 0.478 | 15.223 | 0.232 | 0.110 |
| Intra-Fungal -H (Huishui) | 159 | 1000/96.1% | 41/3.9% | 0.471 | 13.157 | 0.272 | 0.083 |
| Intra-Fungal -D (Guiyang) | 138 | 1000/94.6% | 57/5.4% | 0.500 | 15.319 | 0.282 | 0.112 |
| Intra-Fungal -D (Huishui) | 148 | 1000/97.7% | 24/2.3% | 0.510 | 13.838 | 0.322 | 0.094 |
| Intra-Fungal-H | 123 | 730/100% | 0/0% | 0.486 | 11.87 | 0.269 | 0.097 |
| Intra-Fungal-D | 132 | 1000/99% | 10/1% | 0.568 | 15.303 | 0.317 | 0.117 |
| Inter-H (Guiyang) | 425 | 1000/50% | 1000/50% | 0.337 | 9.412 | 0.492 | 0.022 |
| Inter-H (Huishui) | 470 | 1000/50% | 1000/50% | 0.372 | 8.511 | 0.526 | 0.018 |
| Inter-D (Guiyang) | 377 | 1000/50% | 1000/50% | 0.256 | 10.610 | 0.399 | 0.028 |
| Inter-D (Huishui) | 508 | 1000/50% | 1000/50% | 0.309 | 7.874 | 0.574 | 0.016 |
| Interkingdom-H | 400 | 1000/57.2% | 747/42.8% | 0.404 | 8.73 | 0.535 | 0.022 |
| Interkingdom-D | 378 | 1000/65.1% | 535/34.9% | 0.362 | 8.116 | 0.524 | 0.022 |

H: healthy; D: *Fusarium* wilt disease.

**Table S11** Taxonomic composition of bacterial phylum and fungal class between healthy and diseased intra- and interkingdom networks.

| Networks | Proportion of bacterial phylum and fungal class % | | | | | | | |
| --- | --- | --- | --- | --- | --- | --- | --- | --- |
| Bacterial phylum | Proteobacteria | Actinobacteria | Firmicutes | Bacteroidetes | Chloroflexi | Acidobacteria | Others |  |
| Bacterial-H (Guiyang) Bacterial-H (Huishui) Bacterial-D (Guiyang) Bacterial-D (Huishui)  Bacterial-H  Bacterial-D | 39.8%  39.4%  37.6%  40.2%  39.15%  38.27% | 20.5%  20.2%  24.4%  24.1%  21.42%  24.5% | 13.5%  13.8%  13.7%  14.5%  13.32%  12.44% | 8.11%  6.53&  7.67%  6.58%  7.14%  5.86% | 6.39%  5.74%  6.39%  3.8%  5.36%  5.56% | 3.93%  4.18%  2.3%  3.04%  3.87%  3.09% | 7.77%  10.18%7.94%  7.78%  9.74%  10.28% |  |
| Fungal Class | Sordariomycetes | Eurotiomycetes | Dothideomycetes | Agariomycetes | Tremellomycetes | Pezizomycetes | Leotiomycetes | Others |
| Fungal-H (Guiyang)  Fungal-H (Huishui)  Fungal-D (Guiyang)  Fungal-D (Huishui)  Fungal-H  Fungal-D | 42.3%  40.6%  41.4%  41.2%  43. 9%  46.52% | 12.3%  13.7%  15.3%  13.8%  16.26%  15.9% | 14.2%  19.5%  12.6%  14.1%  12.28%  8.33% | 9.35%  8.81%  6.52%  8.78%  6.55%  7.58% | 7.19%  3.14%  6.52%  4.05%  5.69%  5.3% | 3.6%  1.89%  3.62%  4.05%  4.88%  4.55% | 2.16%  3.14%  3.64%  4.05%  2.44%  3.79% | 8.9%  9.22%  10.4%  9.97%  8%  8.03% |
| Interkingdom | Bacteria node | Fungi node |  |  |  |  |  |  |
| H (Guiyang)  H (Huishui)  D (Guiyang)  D (Huishui) | 76%  81.8%  66.4%  74.5% | 24%  18.2%  33.6%  25.5% |  |  |  |  |  |  |

**Table S12** The taxonomic position of top 10 hubs in intra- and interkingdom networks.

| ZOTUs in intra-kingdom networks | Taxonomic position |
| --- | --- |
| BZOTU189 | Bacteria--Actinobacteria--Actinobacteria1--Micrococcales--Microbacteriaceae--Microbacterium |
| BZOTU916 | Bacteria--Proteobacteria--Alphaproteobacteria--Rhizobiales--Rhizobiaceae--Aureimonas |
| BZOTU871 | Bacteria--Proteobacteria--Alphaproteobacteria--Rhizobiales--Beijerinckiaceae--Methylobacterium |
| BZOTU140 | Bacteria--Actinobacteria--Actinobacteria1--Kineosporiales--Kineosporiaceae--Kineococcus |
| BZOTU1289 | Bacteria--Proteobacteria--Gammaproteobacteria--Enterobacteriales--Enterobacteriaceae--Escherichia-Shigella |
| BZOTU515 | Bacteria--Chloroflexi--JG30-KF-CM66--uncultured soil bacterium--uncultured soil bacteriumcross196--uncultured soil bacterium1--uncultured soil bacterium2 |
| BZOTU1116 | Bacteria--Proteobacteria--Deltaproteobacteria--RCP2-54--uncultured bacterium--uncultured bacteriumcross430--uncultured bacterium1-- |
| BZOTU1222 | Bacteria--Proteobacteria--Gammaproteobacteria--Betaproteobacteriales--Nitrosomonadaceae--DSSD61 |
| BZOTU142 | Bacteria--Actinobacteria--Actinobacteria1--Kineosporiales--Kineosporiaceae--Pseudokineococcus |
| BZOTU1346 | Bacteria--Proteobacteria--Gammaproteobacteria--Salinisphaerales--Solimonadaceae--Polycyclovorans |
| BZOTU1029 | Bacteria--Proteobacteria--Deltaproteobacteria--Bdellovibrionales--Bdellovibrionaceae--OM27 clade |
| BZOTU1291 | Bacteria--Proteobacteria--Gammaproteobacteria--Enterobacteriales--Enterobacteriaceae--Pantoea |
| BZOTU956 | Bacteria--Proteobacteria--Alphaproteobacteria--Rhizobiales--Xanthobacteraceae--uncultured--unculturedcross343 |
| BZOTU935 | Bacteria--Proteobacteria--Alphaproteobacteria--Rhizobiales--Rhizobiales Incertae Sedis--Bauldia |
| BZOTU225 | Bacteria--Actinobacteria--Actinobacteria1--Micromonosporales--Micromonosporaceae--Dactylosporangium |
| BZOTU948 | Bacteria--Proteobacteria--Alphaproteobacteria--Rhizobiales--Xanthobacteraceae--Pseudolabrys |
| BZOTU1111 | Bacteria--Proteobacteria--Deltaproteobacteria--Oligoflexales--0319-6G20--uncultured soil bacterium--uncultured soil bacteriumcross427-- |
| BZOTU322 | Bacteria--Actinobacteria--Thermoleophilia--Solirubrobacterales--67-14--uncultured bacterium--uncultured bacteriumcross122 |
| FZOTU378 | Fungi--Ascomycota--Sordariomycetes--Microascales--Microascaceae--Scedosporium |
| FZOTU246 | Fungi--Ascomycota--Pezizomycetes--Pezizales--Pyronemataceae--Pseudaleuria |
| FZOTU393 | Fungi--Ascomycota--Sordariomycetes--Sordariales--Chaetomiaceae--Melanocarpus |
| FZOTU415 | Fungi--Ascomycota--Sordariomycetes--Sordariales--Sordariales_fam_Incertae_sedis--Remersonia |
| FZOTU185 | Fungi--Ascomycota--Eurotiomycetes--Onygenales--unidentified--unidentifiedcross33--unidentified1 |
| FZOTU388 | Fungi--Ascomycota--Sordariomycetes--Sordariales--Chaetomiaceae--Acrophialophora |
| FZOTU411 | Fungi--Ascomycota--Sordariomycetes--Sordariales--Sordariaceae--unidentified--unidentifiedcross79 |
| FZOTU640 | Fungi--Chytridiomycota--Chytridiomycetes--unidentified--unidentifiedcross129--unidentified1--unidentified2 |
| FZOTU397 | Fungi--Ascomycota--Sordariomycetes--Sordariales--Chaetomiaceae--Thielavia |
| FZOTU164 | Fungi--Ascomycota--Eurotiomycetes--Eurotiales--Trichocomaceae--unidentified--unidentifiedcross27 |
| FZOTU214 | Fungi--Ascomycota--Leotiomycetes--Helotiales--Helotiaceae--Tetracladium |
| FZOTU298 | Fungi--Ascomycota--Sordariomycetes--Hypocreales--Bionectriaceae--unidentified--unidentifiedcross60 |
| FZOTU293 | Fungi--Ascomycota--Sordariomycetes--Glomerellales--Plectosphaerellaceae--unidentified--unidentifiedcross59 |
| FZOTU450 | Fungi--Basidiomycota--Agaricomycetes--Agaricales--Agaricaceae--Leucocoprinus |
| FZOTU300 | Fungi--Ascomycota--Sordariomycetes--Hypocreales--Clavicipitaceae--Claviceps |
| FZOTU281 | Fungi--Ascomycota--Sordariomycetes--Diaporthales--Schizoparmaceae--Pilidiella |
| FZOTU314 | Fungi--Ascomycota--Sordariomycetes--Hypocreales--Hypocreaceae--unidentified--unidentifiedcross63 |
| FZOTU143 | Fungi--Ascomycota--Eurotiomycetes--Chaetothyriales--Herpotrichiellaceae--Coniosporium |
| ZOTUs in interkingdom networks | Taxonomic position |
| BZOTU189 | Bacteria--Actinobacteria--Actinobacteria1--Micrococcales--Microbacteriaceae—Microbacterium |
| BZOTU916 | Bacteria--Proteobacteria--Alphaproteobacteria--Rhizobiales--Rhizobiaceae--Aureimonas |
| BZOTU871 | Bacteria--Proteobacteria--Alphaproteobacteria--Rhizobiales--Beijerinckiaceae--Methylobacterium |
| BZOTU140 | Bacteria--Actinobacteria--Actinobacteria1--Kineosporiales--Kineosporiaceae--Kineococcus |
| BZOTU249 | Bacteria--Actinobacteria--Actinobacteria1--Propionibacteriales--Propionibacteriaceae--Cutibacterium |
| BZOTU515 | Bacteria--Chloroflexi--JG30-KF-CM66--uncultured soil bacterium--uncultured soil bacteriumcross196--uncultured soil bacterium1--uncultured soil bacterium2 |
| BZOTU227 | Bacteria--Actinobacteria--Actinobacteria1--Micromonosporales--Micromonosporaceae--Krasilnikovia |
| BZOTU1222 | Bacteria--Proteobacteria--Gammaproteobacteria--Betaproteobacteriales--Nitrosomonadaceae--DSSD61 |
| BZOTU1102 | Bacteria--Proteobacteria--Deltaproteobacteria--Oligoflexales--0319-6G20--metagenome--metagenomecross421 |
| BZOTU1346 | Bacteria--Proteobacteria--Gammaproteobacteria--Salinisphaerales--Solimonadaceae--Polycyclovorans |
| BZOTU1291 | Bacteria--Proteobacteria--Gammaproteobacteria--Enterobacteriales--Enterobacteriaceae--Pantoea |
| BZOTU1029 | Bacteria--Proteobacteria--Deltaproteobacteria--Bdellovibrionales--Bdellovibrionaceae--OM27 clade |
| FZOTU293 | Fungi--Ascomycota--Sordariomycetes--Glomerellales--Plectosphaerellaceae--unidentified--unidentifiedcross570 |
| FZOTU143 | Fungi--Ascomycota--Eurotiomycetes--Chaetothyriales--Herpotrichiellaceae--Coniosporium |
| FZOTU298 | Fungi--Ascomycota--Sordariomycetes--Hypocreales--Bionectriaceae--unidentified--unidentifiedcross571 |
| FZOTU393 | Fungi--Ascomycota--Sordariomycetes--Sordariales--Chaetomiaceae--Melanocarpus |
| FZOTU314 | Fungi--Ascomycota--Sordariomycetes--Hypocreales--Hypocreaceae--unidentified--unidentifiedcross574 |

**Table S13** Numbers of enriched and depleted functional genes (including KO, COG, CAZ, and ResFam profiles) in diseased plant compared with the healthy plant calculated by the LEfSe difference analysis.

| Functional profiles | Compartments | Condition | Number of enriched functions |
| --- | --- | --- | --- |
| KO | Rendo | depleted | 8 |
|  |  | enriched | 3 |
|  | US- epidermis | depleted | 55 |
|  |  | enriched | 69 |
| COG | Rendo | depleted | 1 |
|  |  | enriched | 2 |
|  | US- epidermis | depleted | 15 |
|  |  | enriched | 5 |
| CAZ | Rendo | depleted | 10 |
|  |  | enriched | 10 |
|  | US- epidermis | depleted | 27 |
|  |  | enriched | 15 |
| Resfam | Rendo | depleted | 26 |
|  |  | enriched | 27 |
|  | US- epidermis | depleted | 51 |
|  |  | enriched | 34 |

**Table S14** Functional annotation of differentially abundant genes (top 20) between healthy and diseased plant calculated by LEfSe difference analysis.

| Functional profiles | Functional ID | Description |
| --- | --- | --- |
| COG | COG_M | Cell wall/membrane/ecope biogenesis |
|  | COG_Z | Cytoskeleton |
|  | COG_W | Extracellular structures |
|  | COG_S | Function unknown |
|  | COG_L | Replication, recombination and repair |
|  | COG_A | RNA processing and modification |
|  | COG_K | Transcription |
|  | COG_J | Translation, ribosomal structure and biogenesis |
|  | COG_E | Amino acid transport and metabolism |
|  | COG_G | Carbohydrate transport and metabolism |
|  | COG_C | Energy production and conversion |
|  | COG_P | Inorganic ion transport and metabolism |
|  | COG_I | Lipid transport and metabolism |
|  | COG_Q | Secondary metabolites biosynthesis, transport and catabolism |
|  | COG_T | Signal transduction mechanisms |
|  | COG_V | Defense mechanisms |
|  | COG_O | Posttranslational modification, protein turnover, chaperones |
| ResFam | resfam1 | chloramphenicol efflux pump |
|  | resfam2 | macB: subunit of efflux pump conferring antibiotic resistance |
|  | resfam3 | VanS: trasncriptional regulator of van glycopeptide resistance genes |
|  | resfam4 | VanS: trasncriptional regulator of van glycopeptide resistance genes |
|  | resfam5 | VanS: trasncriptional regulator of van glycopeptide resistance genes |
|  | resfam6 | VanS: transcriptional regulator of van glycopeptide resistance genes |
|  | resfam7 | VanR: transcriptional activator regulating VanA, VanH and VanX |
|  | resfam8 | phoQ: subunit of gene modulating antibiotic efflux |
|  | resfam9 | VanS: trasncriptional regulator of van glycopeptide resistance genes |
|  | resfam10 | baeS: subunit of gene modulating antibiotic efflux |
|  | resfam11 | VanR: transcriptional activator regulating VanA, VanH and VanX |
|  | resfam12 | VanH: D-specific alpha-ketoacid dehydrogenase that synthesizes D-lactate |
|  | resfam13 | VanH: D-specific alpha-ketoacid dehydrogenase that synthesizes D-lactate |
|  | resfam14 | major facilitator superfamily (MFS) antibiotic efflux pump |
|  | resfam15 | chloramphenicol efflux pump |
|  | resfam16 | adeS: gene modulating antibiotic efflux regulating AdeABC |
|  | resfam17 | resistance-nodulation-cell division (RND) antibiotic efflux pump |
|  | resfam18 | resistance-nodulation-cell division (RND) antibiotic efflux pump |
|  | resfam19 | tetracycline resistance ribosomal protection protein: protect RNA-polymerase from tetracycline inhibition |
|  | resfam20 | tetracycline resistance ribosomal protection protein: protect RNA-polymerase from tetracycline inhibition |
|  | resfam21 | Cass C beta-lactamases |
|  | resfam22 | VanS: trasncriptional regulator of van glycopeptide resistance genes |
|  | resfam23 | mprF: peptide antibiotic resistance gene |
|  | resfam24 | VanR: transcriptional activator regulating VanA, VanH and VanX |
|  | resfam25 | soxR: mutant efflux regulatory protein conferring antibiotic resistance |
|  | resfam26 | baeS: subunit of gene modulating antibiotic efflux |
|  | resfam27 | baeS: subunit of gene modulating antibiotic efflux |
|  | resfam28 | VanS: trasncriptional regulator of van glycopeptide resistance genes |
|  | resfam29 | VanT: membrane bound serine racemase, converting L-serine to D-serine |
|  | resfam30 | adeS: gene modulating antibiotic efflux regulating AdeABC |
|  | resfam31 | chloramphenicol phosphotransferase (CPT) |
|  | resfam32 | Fluoroquinolone Resistant DNA Topoisomerase |
|  | resfam33 | Fluoroquinolone Resistant DNA Topoisomerase |
|  | resfam34 | mexA: membrane fusion protein of the MexAB-OprM multidrug efflux complex |
|  | resfam35 | resistance-nodulation-cell division (RND) antibiotic efflux pump |
|  | resfam36 | resistance-nodulation-cell division (RND) antibiotic efflux pump |
|  | resfam37 | adeC-adeK-oprM: outer membrane factor the multidrug efflux complex |
|  | resfam38 | adeC-adeK-oprM: outer membrane factor the multidrug efflux complex |
|  | resfam39 | TE_inactivation |
|  | resfam40 | TE_inactivation |
| KO | K18667 | activating signal cointegrator complex subunit 2 |
|  | K01768 | adenylate cyclase [EC:4.6.1.1] |
|  | K13735 | adhesin/invasin |
|  | K05349 | beta-glucosidase [EC:3.2.1.21] |
|  | K08504 | blocked early in transport 1 |
|  | K08057 | calreticulin |
|  | K09250 | cellular nucleic acid-binding protein |
|  | K02256 | cytochrome c oxidase subunit 1 [EC:1.9.3.1] |
|  | K01179 | endoglucanase [EC:3.2.1.4] |
|  | K00123 | formate dehydrogenase major subunit [EC:1.2.1.2] |
|  | K02132 | F-type H+-transporting ATPase subunit alpha |
|  | K04333 | LuxR family transcriptional regulator, csgAB operon transcriptional regulatory protein |
|  | K00962 | polyribonucleotide nucleotidyltransferase [EC:2.7.7.8] |
|  | K02004 | putative ABC transport system permease protein |
|  | K07478 | putative ATPase |
|  | K03088 | RNA polymerase sigma-70 factor, ECF subfamily |
|  | K00384 | thioredoxin reductase (NADPH) [EC:1.8.1.9] |
|  | K01153 | type I restriction enzyme, R subunit [EC:3.1.21.3] |
|  | K03199 | type IV secretion system protein VirB4 |
|  | K07263 | zinc protease [EC:3.4.24.-] |
|  | K01083 | 3-phytase [EC:3.1.3.8] |
|  | K01113 | alkaline phosphatase D [EC:3.1.3.1] |
|  | K15539 | cytoskeleton protein RodZ |
|  | K01727 | hyaluronate lyase [EC:4.2.2.1] |
|  | K04333 | LuxR family transcriptional regulator, csgAB operon transcriptional regulatory protein |
|  | K14647 | minor extracellular serine protease Vpr [EC:3.4.21.-] |
|  | K02405 | RNA polymerase sigma factor for flagellar operon FliA |
|  | K07165 | transmembrane sensor |
|  | K07484 | transposase |
|  | K14415 | tRNA-splicing ligase RtcB [EC:6.5.1.3] |
|  | K01153 | type I restriction enzyme, R subunit [EC:3.1.21.3] |
| CAZ | GT39 | Dol-P-Man: protein alpha-mannosyltransferase (EC 2.4.1.109) |
|  | GH32 | invertase (EC 3.2.1.26); endo-inulinase (EC 3.2.1.7); beta-2,6-fructan 6-levanbiohydrolase (EC 3.2.1.64); endo-levanase (EC 3.2.1.65); exo-inulinase (EC 3.2.1.80); fructan beta-(2,1)-fructosidase/1-exohydrolase (EC 3.2.1.153); fructan beta-(2,6)-fructosidase/6-exohydrolase (EC 3.2.1.154); sucrose:sucrose 1-fructosyltransferase (EC 2.4.1.99); fructan:fructan 1-fructosyltransferase (EC 2.4.1.100); sucrose:fructan 6-fructosyltransferase (EC 2.4.1.10); fructan:fructan 6G-fructosyltransferase (EC 2.4.1.243); levan fructosyltransferase (EC 2.4.1.-); [retaining] sucrose:sucrose 6-fructosyltransferase (6-SST) (EC 2.4.1.-); cycloinulo-oligosaccharide fructanotransferase (EC 2.4.1.-) |
|  | CBM5 | Modules of approx. 60 residues found in bacterial enzymes. Chitin-binding described in several cases. Distantly related to the CBM12 family. |
|  | GT31 | N-acetyllactosaminide beta-1,3-N-acetylglucosaminyltransferase (EC 2.4.1.149); Glycoprotein-N-acetylgalactosamine 3-beta-galactosyltransferase (EC 2.4.1.122); fucose-specific beta-1,3-N-acetylglucosaminyltransferase (EC 2.4.1.-); globotriosylceramide beta-1,3-GalNAc transferase (EC 2.4.1.79); chondroitin synthase (beta-1,3-GlcUA and beta-1,4-GalNAc transferase (EC 2.4.1.175); chondroitin beta-1,3-glucuronyltransferase (EC 2.4.1.226); chondroitin beta-1,4-N-acetylgalactosaminyltransferase (EC 2.4.1.-); UDP-Gal: beta-galactosylxylosylprotein beta-1,3-galactosyltransferase (EC 2.4.1.134); UDP-GlcNAc: O-fucosylpeptide beta-1,3-N-acetylglucosaminyltransferase (EC 2.4.1.222) |
|  | GH88 | d-4,5-unsaturated beta-glucuronyl hydrolase (EC 3.2.1.-) |
|  | GT89 | beta-D-arabinofuranosyl-1-monophosphoryldecaprenol : arabinan beta-1,2-arabinofuranosyltransferase (EC 2.4.2.-) |
|  | AA10 | AA10 (formerly CBM33) proteins are copper-dependent lytic polysaccharide monooxygenases (LPMOs); some proteins have been shown to act on chitin, others on cellulose; |
|  | GH36 | alpha-galactosidase (EC 3.2.1.22); alpha-N-acetylgalactosaminidase (EC 3.2.1.49); stachyose synthase (EC 2.4.1.67); raffinose synthase (EC 2.4.1.82) |
|  | GT47 | heparan beta-glucuronyltransferase (EC 2.4.1.225); xyloglucan beta-galactosyltransferase (EC 2.4.1.-); heparan synthase (EC 2.4.1.-); arabinan alpha-L-arabinosyltransferase (EC 2.4.2.-). |
|  | GH1 | beta-glucosidase (EC 3.2.1.21); beta-galactosidase (EC 3.2.1.23); beta-mannosidase (EC 3.2.1.25); beta-glucuronidase (EC 3.2.1.31); beta-xylosidase (EC 3.2.1.37); beta-D-fucosidase (EC 3.2.1.38); phlorizin hydrolase (EC 3.2.1.62); exo-beta-1,4-glucanase (EC 3.2.1.74); 6-phospho-beta-galactosidase (EC 3.2.1.85); 6-phospho-beta-glucosidase (EC 3.2.1.86); strictosidine beta-glucosidase (EC 3.2.1.105); lactase (EC 3.2.1.108); amygdalin beta-glucosidase (EC 3.2.1.117); prunasin beta-glucosidase (EC 3.2.1.118); vicianin hydrolase (EC 3.2.1.119); raucaffricine beta-glucosidase (EC 3.2.1.125); thioglucosidase (EC 3.2.1.147); beta-primeverosidase (EC 3.2.1.149); isoflavonoid 7-O-beta-apiosyl-beta-glucosidase (EC 3.2.1.161); ABA-specific beta-glucosidase (EC 3.2.1.175); DIMBOA beta-glucosidase (EC 3.2.1.182); beta-glycosidase (EC 3.2.1.-); hydroxyisourate hydrolase (EC 3.-.-.-) |
|  | GT26 | UDP-ManNAcA: beta-N-acetyl mannosaminuronyltransferase (EC 2.4.1.-); UDP-ManNAc: beta-N-acetyl-mannosaminyltransferase (EC 2.4.1.-); UDP-Glc: beta-1,4-glucosyltransferase (EC 2.4.1.-); beta-1,4-galactosyltransferase (EC 2.4.1.-) |
|  | GT25 | lipopolysaccharide beta-1,4-galactosyltransferase (EC 2.4.1.-); beta-1,3-glucosyltransferase (EC 2.4.1.-); beta-1,2-glucosyltransferase (EC 2.4.1.-); beta-1,2-galactosyltransferase (EC 2.4.1.-); LPS beta-1,4-galactosyltransferase (EC 2.4.1.-); occidiofungin beta-xylosyltransferase (EC 2.4.2.-); UDP-Gal:procollagen beta-galactosyltransferase (EC 2.4.1.50) |
|  | GT27 | polypeptide alpha-N-acetylgalactosaminyltransferase (EC 2.4.1.41) |
|  | GH8 | chitosanase (EC 3.2.1.132); cellulase (EC 3.2.1.4); licheninase (EC 3.2.1.73); endo-1,4-beta-xylanase (EC 3.2.1.8); reducing-end-xylose releasing exo-oligoxylanase (EC 3.2.1.156) |
|  | GH127 | beta-L-arabinofuranosidase (EC 3.2.1.185); 3-C-carboxy-5-deoxy-L-xylose (aceric acid) hydrolase (EC 3.2.1.-) |
|  | GT66 | dolichyl-diphosphooligosaccharide?€”protein glycotransferase (EC 2.4.99.18); undecaprenyl-diphosphooligosaccharide?€”protein glycotransferase (EC 2.4.99.19) |
|  | CBM6 | Modules of approx. 120 residues. The cellulose-binding function has been demonstrated in one case on amorphous cellulose and beta-1,4-xylan. Some of these modules also bind beta-1,3-glucan, beta-1,3-1,4-glucan, and beta-1,4-glucan. |
|  | GH99 | glycoprotein endo-alpha-1,2-mannosidase (EC 3.2.1.130); mannan endo-1,2-alpha-mannanase (3.2.1.-) |
|  | GH66 | cycloisomaltooligosaccharide glucanotransferase (EC 2.4.1.248); dextranase (EC 3.2.1.11). |
|  | GT85 | beta-D-arabinofuranosyl monophosphoryldecaprenol: galactan alpha-D-arabinofuranosyltransferase (EC 2.4.2.-) |
|  | GH5 | endo-beta-1,4-glucanase / cellulase (EC 3.2.1.4); endo-beta-1,4-xylanase (EC 3.2.1.8); beta-glucosidase (EC 3.2.1.21); beta-mannosidase (EC 3.2.1.25); beta-glucosylceramidase (EC 3.2.1.45); glucan beta-1,3-glucosidase (EC 3.2.1.58); licheninase (EC 3.2.1.73); exo-beta-1,4-glucanase / cellodextrinase (EC 3.2.1.74); glucan endo-1,6-beta-glucosidase (EC 3.2.1.75); mannan endo-beta-1,4-mannosidase (EC 3.2.1.78); cellulose beta-1,4-cellobiosidase (EC 3.2.1.91); steryl beta-glucosidase (EC 3.2.1.104); endoglycoceramidase (EC 3.2.1.123); chitosanase (EC 3.2.1.132); beta-primeverosidase (EC 3.2.1.149); xyloglucan-specific endo-beta-1,4-glucanase (EC 3.2.1.151); endo-beta-1,6-galactanase (EC 3.2.1.164); hesperidin 6-O-alpha-L-rhamnosyl-beta-glucosidase (EC 3.2.1.168); beta-1,3-mannanase (EC 3.2.1.-); arabinoxylan-specific endo-beta-1,4-xylanase (EC 3.2.1.-); mannan transglycosylase (EC 2.4.1.-) |
|  | GH3 | beta-glucosidase (EC 3.2.1.21); xylan 1,4-beta-xylosidase (EC 3.2.1.37); beta-glucosylceramidase (EC 3.2.1.45); beta-N-acetylhexosaminidase (EC 3.2.1.52); alpha-L-arabinofuranosidase (EC 3.2.1.55); glucan 1,3-beta-glucosidase (EC 3.2.1.58); glucan 1,4-beta-glucosidase (EC 3.2.1.74); isoprimeverose-producing oligoxyloglucan hydrolase (EC 3.2.1.120); coniferin beta-glucosidase (EC 3.2.1.126); exo-1,3-1,4-glucanase (EC 3.2.1.-); beta-N-acetylglucosaminide phosphorylases (EC 2.4.1.-) |
|  | GH31 | alpha-glucosidase (EC 3.2.1.20); alpha-galactosidase (EC 3.2.1.22); alpha-mannosidase (EC 3.2.1.24); alpha-1,3-glucosidase (EC 3.2.1.84); sucrase-isomaltase (EC 3.2.1.48) (EC 3.2.1.10); alpha-xylosidase (EC 3.2.1.177); alpha-glucan lyase (EC 4.2.2.13); isomaltosyltransferase (EC 2.4.1.-); oligosaccharide alpha-1,4-glucosyltransferase (EC 2.4.1.161); sulfoquinovosidase (EC 3.2.1.-) |
|  | GH95 | alpha-L-fucosidase (EC 3.2.1.51); alpha-1,2-L-fucosidase (EC 3.2.1.63); alpha-L-galactosidase (EC 3.2.1.-) |
|  | GH68 | levansucrase (EC 2.4.1.10); beta-fructofuranosidase (EC 3.2.1.26); inulosucrase (EC 2.4.1.9). |
|  | GH43 | beta-xylosidase (EC 3.2.1.37); alpha-L-arabinofuranosidase (EC 3.2.1.55); arabinanase (EC 3.2.1.99); xylanase (EC 3.2.1.8); galactan 1,3-beta-galactosidase (EC 3.2.1.145); alpha-1,2-L-arabinofuranosidase (EC 3.2.1.-); exo-alpha-1,5-L-arabinofuranosidase (EC 3.2.1.-); [inverting] exo-alpha-1,5-L-arabinanase (EC 3.2.1.-); beta-1,3-xylosidase (EC 3.2.1.-) |
|  | GH19 | chitinase (EC 3.2.1.14); lysozyme (EC 3.2.1.17) |
|  | GH19 | chitinase (EC 3.2.1.14); lysozyme (EC 3.2.1.17) |
|  | GH121 | beta-L-arabinobiosidase (EC 3.2.1.-) |
|  | PL11 | rhamnogalacturonan endolyase (EC 4.2.2.23); rhamnogalacturonan exolyase (EC 4.2.2.24) |
|  | GT1 | UDP-glucuronosyltransferase (EC 2.4.1.17); zeatin O-beta-xylosyltransferase (EC 2.4.2.40); 2-hydroxyacylsphingosine 1-beta-galactosyltransferase (EC 2.4.1.45); N-acylsphingosine galactosyltransferase (EC 2.4.1.47); flavonol 3-O-glucosyltransferase (EC 2.4.1.91); anthocyanidin 3-O-glucosyltransferase (EC 2.4.1.115); sinapate 1-glucosyltransferase (EC 2.4.1.120); indole-3-acetate beta-glucosyltransferase (EC 2.4.1.121); flavonol L-rhamnosyltransferase (EC 2.4.1.159); sterol glucosyltransferase (EC 2.4.1.173); UDP-Glc: 4-hydroxybenzoate 4-O-beta-glucosyltransferase (EC 2.4.1.194); zeatin O-beta-glucosyltransferase (EC 2.4.1.203); limonoid glucosyltransferase (EC 2.4.1.210); UDP-GlcA: baicalein 7-O-beta-glucuronosyltransferase (EC 2.4.1.253); UDP-Glc: chalcone 4?€?-O-beta-glucosyltransferase (EC 2.4.1.286); ecdysteroid UDP-glucosyltransferase (EC 2.4.1.-); salicylic acid beta-glucosyltransferase (EC 2.4.1.-); anthocyanin 3-O-galactosyltransferase (EC 2.4.1.-); anthocyanin 5-O-glucosyltransferase (EC 2.4.1.-); dTDP-beta-2-deoxy-L-fucose: alpha-L-2-deoxyfucosyltransferase (EC 2.4.1.-); UDP-beta-L-rhamnose: alpha-L-rhamnosyltransferase (EC 2.4.1.-); zeaxanthin glucosyltransferase (EC 2.4.1.-) |
|  | GT51 | murein polymerase (EC 2.4.1.129). |
|  | CBM18 | Modules of approx. 40 residues. The chitin-binding function has been demonstrated in many cases. These modules are found attached to a number of chitinase catalytic domains, but also in non-catalytic proteins either in isolation or as multiple repeats. |
|  | CBM48 | Modules of approx. 100 residues with glycogen-binding function, appended to GH13 modules. Also found in the beta subunit (glycogen-binding) of AMP-activated protein kinases (AMPK) |
|  | GH38 | alpha-mannosidase (EC 3.2.1.24); mannosyl-oligosaccharide alpha-1,2-mannosidase (EC 3.2.1.113); mannosyl-oligosaccharide alpha-1,3-1,6-mannosidase (EC 3.2.1.114); alpha-2-O-mannosylglycerate hydrolase (EC 3.2.1.170); mannosyl-oligosaccharide alpha-1,3-mannosidase (EC 3.2.1.-) |
|  | GT51 | murein polymerase (EC 2.4.1.129). |
|  | GT35 | glycogen or starch phosphorylase (EC 2.4.1.1). |
|  | GT39 | Dol-P-Man: protein alpha-mannosyltransferase (EC 2.4.1.109) |
|  | GH36 | alpha-galactosidase (EC 3.2.1.22); alpha-N-acetylgalactosaminidase (EC 3.2.1.49); stachyose synthase (EC 2.4.1.67); raffinose synthase (EC 2.4.1.82) |
|  | GH33 | sialidase or neuraminidase (EC 3.2.1.18); trans-sialidase (EC 2.4.1.-); anhydrosialidase (EC 4.2.2.15); Kdo hydrolase (EC 3.2.1.-); 2-keto-3-deoxynononic acid hydrolase / KDNase (EC 3.2.1.-) |
